# Loss of CpFTSY Reduces Photosynthetic Performance and Affects Insertion of PsaC of PSI in Diatoms

**DOI:** 10.1093/pcp/pcad014

**Published:** 2023-02-22

**Authors:** Marianne Nymark, Giovanni Finazzi, Charlotte Volpe, Manuel Serif, Davi de Miranda Fonseca, Animesh Sharma, Nicolas Sanchez, Amit Kumar Sharma, Felicity Ashcroft, Ralph Kissen, Per Winge, Atle Magnar Bones

**Affiliations:** Department of Biology, Norwegian University of Science and Technology, Trondheim N-7491, Norway; Department of Fisheries and New Biomarine Industry, SINTEF Ocean, Trondheim 7010, Norway; Cell & Plant Physiology Laboratory, Université Grenoble Alpes, CNRS, CEA, INRAE, IRIG, LPCV, Grenoble 38000, France; Department of Biology, Norwegian University of Science and Technology, Trondheim N-7491, Norway; Department of Fisheries and New Biomarine Industry, SINTEF Ocean, Trondheim 7010, Norway; Department of Biology, Norwegian University of Science and Technology, Trondheim N-7491, Norway; Department of Clinical and Molecular Medicine, Norwegian University of Science and Technology, NTNU, Trondheim N-7491, Norway; Proteomics and Modomics Experimental Core Facility (PROMEC), NTNU and Central Administration, St. Olavs Hospital, The University Hospital in Trondheim, Trondheim N-7491, Norway; Department of Clinical and Molecular Medicine, Norwegian University of Science and Technology, NTNU, Trondheim N-7491, Norway; Proteomics and Modomics Experimental Core Facility (PROMEC), NTNU and Central Administration, St. Olavs Hospital, The University Hospital in Trondheim, Trondheim N-7491, Norway; Department of Chemistry, Norwegian University of Science and Technology, Trondheim N-7491, Norway; Department of Biology, Norwegian University of Science and Technology, Trondheim N-7491, Norway; Department of Biology, Norwegian University of Science and Technology, Trondheim N-7491, Norway; Department of Biology, Norwegian University of Science and Technology, Trondheim N-7491, Norway; Department of Biology, Norwegian University of Science and Technology, Trondheim N-7491, Norway; Department of Biology, Norwegian University of Science and Technology, Trondheim N-7491, Norway

**Keywords:** CpSRP pathway, CRISPR, Diatoms, *Phaeodactylum tricornutum*, Photosynthesis, Thylakoid protein insertion

## Abstract

The chloroplast signal recognition particle (CpSRP) receptor (CpFTSY) is a component of the CpSRP pathway that post-translationally targets light-harvesting complex proteins (LHCPs) to the thylakoid membranes in plants and green algae containing chloroplasts derived from primary endosymbiosis. In plants, CpFTSY also plays a major role in the co-translational incorporation of chloroplast-encoded subunits of photosynthetic complexes into the thylakoids. This role has not been demonstrated in green algae. So far, its function in organisms with chloroplasts derived from secondary endosymbiotic events has not been elucidated. Here, we report the generation and characterization of mutants lacking CpFTSY in the diatom *Phaeodactylum tricornutum.* We found that this protein is not involved in inserting LHCPs into thylakoid membranes, indicating that the post-translational part of the CpSRP pathway is not active in this group of microalgae. The lack of CpFTSY caused an increased level of photoprotection, low electron transport rates, inefficient repair of photosystem II (PSII), reduced growth, a strong decline in the PSI subunit PsaC and upregulation of proteins that might compensate for a non-functional co-translational CpSRP pathway during light stress conditions. The phenotype was highly similar to the one described for diatoms lacking another component of the co-translational CpSRP pathway, the CpSRP54 protein. However, in contrast to *cpsrp54* mutants, only one thylakoid membrane protein, PetD of the Cyt*b*_6_*f* complex, was downregulated in *cpftsy*. Our results point to a minor role for CpFTSY in the co-translational CpSRP pathway, suggesting that other mechanisms may partially compensate for the effect of a disrupted CpSRP pathway.

## Introduction

Diatoms are among the ecologically most significant groups of microorganisms on the Earth, contributing 15–20% to the total primary photosynthetic productivity on the planet (Nelson et al. 1995, Armbrust 2009). Besides its primary ecological relevance, this phytoplankton group also promises a multitude of potential biotechnological applications, and the generation of diatom strains that are more suitable for growth in photobioreactors is of great interest (Bozarth et al. 2009, Levitan et al. 2014, Butler et al. 2020). One approach to improve photosynthetic productivity in a mass culture is to reduce its optical density to favor a more homogeneous light utilization and avoid light dissipation as thermal energy (Melis 2009, Vecchi et al. 2020). This can in principle be done by decreasing the amount of light-harvesting complex proteins (LHCPs) and thereby the pigment content per cell, thus ensuring that light availability is not a limiting factor for growth under conditions of high cell densities and high light (HL) intensities (Melis 2009, Kirst and Melis 2014, Vecchi et al. 2020). To test this hypothesis, we targeted the components of the chloroplast signal recognition particle (CpSRP) pathway in diatoms. This pathway is important for the insertion of chloroplast proteins, including LHCPs, into the thylakoid membranes of plants and green algae (Sundberg et al. 1997, Amin et al., 1999, Göhre et al. 2006, Tzvetkova-Chevolleau et al. 2007, Kirst et al. 2012a, 2012b, Jeong et al. 2017). In the green lineage, the post-translational transport of nucleus-encoded LHCPs from the chloroplast membrane to the thylakoid membrane depends on binding to the CpSRP components, comprising the LHCP-specific chaperone CpSRP43 and the CpSRP54 GTPase. This transit complex is recognized by CpFTSY, the receptor of CpSRP54, that directs the complex to the CpSRP insertase ALBINO3 (ALB3) localized in the thylakoid membrane (Kirst and Melis 2014, Ziehe et al. 2018). Both CpSRP54 and CpFTSY have an N-terminal (N) binding domain and a GTPase (G) domain, and binding and hydrolysis of GTP through their NG domains are essential for the assembly of the transit complex with CpFTSY and the disassembly and release of the cargo protein at the thylakoid membrane, respectively (Tu et al. 1999, Yuan et al. 2002, Yang et al. 2011, Ziehe et al. 2018). Loss of any of the components of the CpSRP pathway in plants and green algae decreases the level of various LHCPs and consequently also the amount of pigments per cell (Sundberg et al. 1997, Amin et al., 1999, Göhre et al. 2006, Tzvetkova-Chevolleau et al. 2007, Kirst et al. 2012a, 2012b, Jeong et al. 2017). As a result of a smaller light-harvesting antenna, plant and green algae CpSRP pathway mutants display a pale green color. In plants, the CpSRP pathway also co-translationally targets chloroplast-encoded subunits of the photosynthetic apparatus to thylakoid membranes (Sundberg et al. 1997, Amin et al., 1999, Tzvetkova-Chevolleau et al. 2007, Walter et al. 2015, Hristou et al. 2019). The co-translational part of the CpSRP pathway is independent of CpSRP43, whereas CpSRP54, CpFTSY and ALB3 have dual roles in the insertion of chloroplast proteins into thylakoid membranes (Ziehe et al. 2018). In the co-translational pathway, CpSRP54 does not interact with CpSRP43, but rather with ribosome nascent chain complexes, whereas the CpSRP54 receptor CpFTSY favors binding of translating ribosomes to thylakoid membranes (Walter et al. 2015, Hristou et al. 2019). The involvement of CpSRP54 and CpFTSY in this process is still uncertain in green algae (Kirst et al. 2012a, Kirst and Melis 2014, Jeong et al. 2017), whereas one of the two green algae ALB3 homologs (ALB3.2) is essential for the assembly of the photosystems (Göhre et al. 2006).

CpSRP43 is absent from diatom genomes (Träger et al. 2012), but we have identified diatom homologs of ALB3 (ALB3a and ALB3b), CpSRP54 and CpFTSY (Nymark et al. 2019, 2021). The three latter proteins are also found in red algae, haptophytes and the *Stramenopila*, *Alveolata* and *Rhizaria* (SAR) clade, whereas CpSRP43 proteins seem to be restricted to the green lineage (Nymark et al. 2021). Outside the green lineage, functional data for CpSRP pathway proteins are only available for diatom ALB3 and CpSRP54 proteins. The genes encoding ALB3a, ALB3b, CpSRP54 and CpFTSY in the model diatom *Phaeodactylum tricornutum* have been targeted for clustered regularly interspaced short palindromic repeats (CRISPR)/CRISPR-associated protein 9 (Cas9) gene editing (Nymark et al. 2019, 2021). No viable bi-allelic knock-out (KO) mutants could be obtained for *ALB3a*, indicating that this is an essential gene, whereas a role for the product of *ALB3b* in the insertion of LHCPs into thylakoid membrane proteins has been confirmed (Nymark et al. 2019). *Alb3b* KO cells were green instead of golden brown due to a strong reduction in the LHCP and light-harvesting pigment content (Nymark et al. 2019). This phenotype likely stems from both a decrease of the carotenoid fucoxanthin (Fx), which is responsible for the brown color of diatoms, and a change in the protein environment in the antenna, which affects the absorption properties of Fx (Gundermann and Büchel 2014, Nymark et al. 2019, Sharma et al. 2021). Despite the lower pigment content, the *alb3b* lines are of little commercial interest because of slow growth at both low light (LL) and HL intensities (Nymark et al. 2019). The negative effect on growth regardless of growth light intensities indicates that the ALB3b insertase has other functions besides insertion of LHCPs. Unlike *alb3b* lines, the loss of diatom CpSRP54 did not cause a decline in LHCPs or light-harvesting pigments, suggesting that the insertion of these proteins is independent of the CpSRP pathway in diatoms. However, lower levels of several chloroplast-encoded subunits of photosynthetic complexes do support a role for the diatom CpSRP54 in the co-translational part of the CpSRP pathway (Nymark et al. 2021). The *cpsrp54*-KO lines also displayed a light-sensitive phenotype likely caused by inefficient repair of photodamaged photosystem II (PSII), resulting in an increased fraction of non-functional PSII during light stress conditions (Nymark et al. 2021).

As mentioned previously, CpFTSY is the receptor of CpSRP54 in plants, where its loss causes a severe chlorotic phenotype (Asakura et al. 2004, Durrett et al. 2006, Tzvetkova-Chevolleau et al. 2007). Maize *cpftsy* mutants (*chloroplast SRP receptor1* mutants) are seedling lethal and contain low levels of several LHCPs and defects in the assembly and accumulation of photosynthetic complexes (Asakura et al. 2004). *Arabidopsis thaliana cpftsy* mutants display a similar phenotype as the maize mutants but are viable despite being chlorotic (Durrett et al. 2006, Tzvetkova-Chevolleau et al. 2007). *A. thaliana cpftsy* has also been linked to the post-translational reduction of iron (Fe)(III) chelatase reductase activity through an unknown mechanism (Durrett et al. 2006). A reduced Fe content in the early stage of plant development has been suggested as a possible explanation for the more severe phenotype of *cpftsy* mutants compared to *cpsrp54* (*chaos*) and *cpsrp43* (*ffc*) since a lack of Fe would affect the functionality of the Fe–sulfur (S) cluster containing complexes of the photosynthetic electron transport chain (Durrett et al. 2006, Tzvetkova-Chevolleau et al. 2007). In contrast, *cpftsy* mutants [*truncated light–harvesting antenna2* (*tla2*) mutants] of the green algae *Chlamydomonas reinhardtii* have a mild phenotype, and it has been proposed that CpFTSY is mainly involved in the post-translational targeting of LHCPs to thylakoid membranes in this group of microalgae (Kirst et al. 2012a). *tla2* mutants display several traits that are desirable for mass culturing like a strong decline in pigment content and a higher photosynthetic productivity than wild-type (WT) cells at high densities and in bright sunlight conditions (Kirst et al. 2012a). So far, the effect of loss of *cpftsy* in diatoms has not previously been described. We generated *cpftsy* KO mutants and exposed them to different light intensities. We compared the responses on pigment content, photosynthetic performance and the amount of chloroplast-localized proteins to the WT. Their phenotype was similar to that of *cpsrp54* mutants, supporting the idea of a divergent mechanism of thylakoid protein insertion in diatoms compared to plants and green algae (Nymark et al. 2021).

## Results and Discussion

### Structure of diatom CpFTSY

**Fig. 1:**
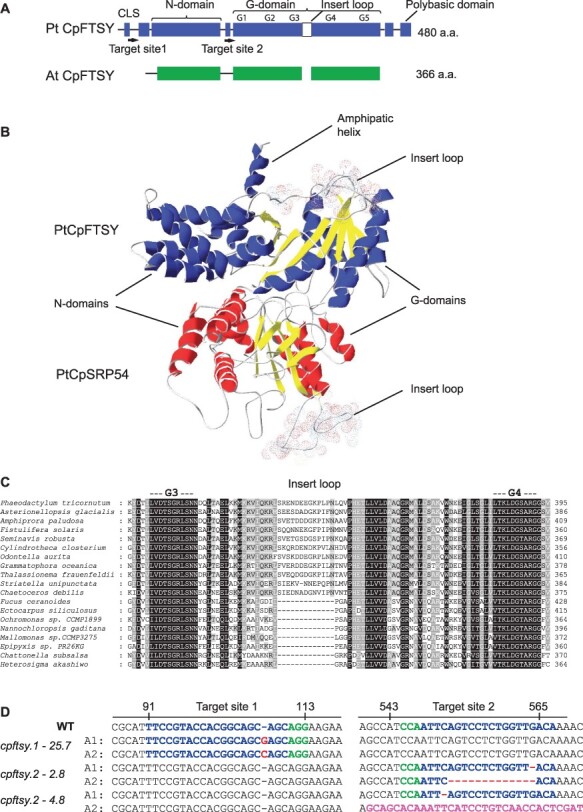
Structural features of CpFTSY and the overview of mutations in *cpftsy* lines. (A) A schematic overview of domains in CpFTSY in*P. tricornutum* (PtCpFTSY) compared to *A. thaliana* (AtCpFTSY). The two sites targeted for CRISPR/Cas9-mediated gene editing are indicated in PtCpFTSY as target sites 1 and 2. (B) A model of the *P. tricornutum* CpFTSY–CpSRP54 heterodimer. The N-terminal amphipathic helix of CpFTSY, the four-helix bundle N-domains, the SRP GTPase–containing domains (G-domains) and the insert loops are indicated in the heterodimer. (C) Protein alignment showing the insert region in the G-domain of CpFTSY in diatoms. (D) An overview of indels in the two alleles (A1 and A2) of *CpFTSY* in *cpftsy.1-25.7* at target site 1 and *cpftsy.2-2.8* and *cpftsy.2-4.8* at target site 2. Blue characters: target sequences; red characters: indels; pink characters: sequence resulting from a chromosomal translocation event and green characters: protospacer adjacent motifs (PAMs). The PAM for target site 2 is located on the reverse strand.

Both CpFTSY and CpSRP54 are members of the SRP GTPase family containing a GTPase domain termed the NG domain through which the two proteins interact and regulate each other’s GTPase activity (Powers and Walter 1995, Jaru-Ampornpan et al. 2007, Chandrasekar et al. 2008, Ziehe et al. 2018). Compared to the primary structure of *A. thaliana* CpFTSY, the *P. tricornutum* CpFTSY is 114 amino acids longer, primarily due to N- and C-terminal conserved domains not found in plant CpFTSYs (**Fig. 1A**, **Supplementary Fig. S1**). The NG domain is highly conserved also in diatom CpFTSY, but diatoms have an additional insert loop of 13 amino acids located between the G-III and G-IV domains (**Fig. 1A****–****C**). We also identified a novel insert loop between the G-I and G-II domains of diatom CpSRP54 proteins (Nymark et al. 2021). In addition to the universally conserved NG domain, the diatom CpFTSY contains an N-terminal chloroplast localization signal and a C-terminal polybasic domain of unknown function similar to the C-terminal domain previously reported for ALB3a (**Fig. 1A**, **Supplementary Figs. S1, S2**) (Nymark et al. 2019). The polybasic domain may modulate membrane association by binding acidic/anionic phospholipids in the chloroplast, such as phosphatidylglycerol and sulfoquinovosyldiacylglycerol (Yalovsky et al. 1999). A model of the CpFTSY and CpSRP54 heterodimer is presented in **Fig. 1B** and reveals that CpFTSY has a protruding amphipathic helix at the N-terminal part of the N-domain that might be important for membrane binding. Similar amphipathic structures containing membrane-binding motifs have been reported for *A. thaliana* CpFTSY and *Escherichia coli* FtsY (Parlitz et al. 2007, Stengel et al. 2007, Marty et al. 2009). Analysis of the conserved N-terminal part of diatom CpFTSY that is not present in plants also identified another potential amphipathic helix, suggesting that diatom CpFTSY may have two membrane-interacting helixes (**Supplementary Fig. S2**). This is also predicted by the AlphaFold2 (Baek et al. 2021, Jumper et al. 2021). The C-terminal polybasic domain is not included in the model.

### Stable *cpftsy* KO lines were created using the CRISPR/Cas9 system

The *CpFTSY* gene was subjected to CRISPR/Cas9-mediated gene editing using two different gRNAs targeting two different regions of the gene (indicated as target sites 1 and 2 in **Fig. 1A**). Vectors containing the Cas9 gene and one of each of the two gRNAs were introduced to the *P. tricornutum* cells using biolistic bombardment. In most cases, gene-editing events resulted in in-frame mutations, but three lines with CRISPR/Cas9-induced frameshift mutations were also identified. Amplification of the *CpFTSY* gene by PCR followed by Sanger sequencing revealed that the *cpftsy.1-25.7* line had a 1-bp insertion at target site 1 in both alleles, whereas the two other lines (*cpftsy.2-2.8* and *cpftsy.2-4.8*) had small deletions at target site 2 (**Fig. 1D**). The small indels responsible for the frameshift mutations caused premature stop codons shortly after the site of the indels, resulting in truncated CpFTSY proteins. The lack of polymorphisms normally present in the *CpFTSY* gene revealed that only one allele had been amplified by PCR for the *cpftsy.2-4.8* mutant (**Supplementary Fig. S3**). No background signal could be seen in the chromatogram for the *cpftsy.2-4.8* sequence (**Supplementary Fig. S3**), indicating that PCR amplification of the other allele was likely prevented by larger insertion, deletion or chromosomal translocation events. MinION nanopore sequencing was additionally performed for all three *cpftsy* lines and revealed that a chromosomal translocation had indeed taken place in the *cpftsy.2-4.8* line at one of the alleles. As indicated in **Fig. 1D**, allele 2 [Chr14 (OU594955.1) pos. 80803] was fused to the promoter/5ʹ untranslated region of a gene encoding an UDP-glucuronate decarboxylase (Phatr3_EG00041) located at Chr23 (OU594964.1) pos. 410424. The nanopore sequencing also confirmed the other mutations in the three *cpftsy* lines revealed by Sanger sequencing of PCR products.

Using biolistic bombardment as a delivery method for the vector encoding the CRISPR components causes the foreign DNA to be randomly integrated into the genome, potentially disturbing random genetic elements. Nanopore sequencing revealed that long repeats of vector DNA had been inserted at two to three different integration sites in each of the *cpftsy* mutant lines. Although the integration of vector DNA caused gene disruption at one allele, nanopore sequencing showed that the other allele was intact. Some of the integrations were inserted into duplicated and highly similar DNA regions, making it impossible to determine the exact chromosome. An overview of the vector DNA integration sites is given in **Supplementary Table S1**. Stable integration of vector DNA causes a constitutive expression of Cas9 (Sharma et al. 2018, George et al. 2020). The CRISPR/Cas9 system used for gene editing of *P. tricornutum* has previously been reported to have the potential to induce off-target gene editing and re-editing at target sites with small indels (1–2 bp) when Cas9 is constitutively expressed (Sharma et al. 2018, 2021). To avoid reporting phenotypic effects that are not a consequence of the lack of CpFTSY but instead caused by any of the reasons listed previously, several precautions were taken: (i) single cells were isolated from independent colonies transformed with the CRISPR/Cas9 vectors to function as a starting point for the three *cpftsy* mutant cultures. The vector DNA had been randomly inserted at different genomic sites in the three independently transformed cells, and potential side effects of the genomic integration are unlikely to be the same. (ii) The gRNAs were carefully designed to have low homology to other genomic loci to minimize the likelihood of inducing off-target gene-editing events. Additionally, the use of two different gRNAs ensured different potential off-target gene-editing events. (iii) The *cpftsy* mutant lines were regularly re-sequenced during the experimental period to ensure that no re-editing events had taken place. At the time of writing, the mutant lines have been in culture and stable for ∼6–7 years.

### Visual examination revealed altered phenotypic traits in the *cpftsy.2-2.8* mutant line

**Fig. 2:**
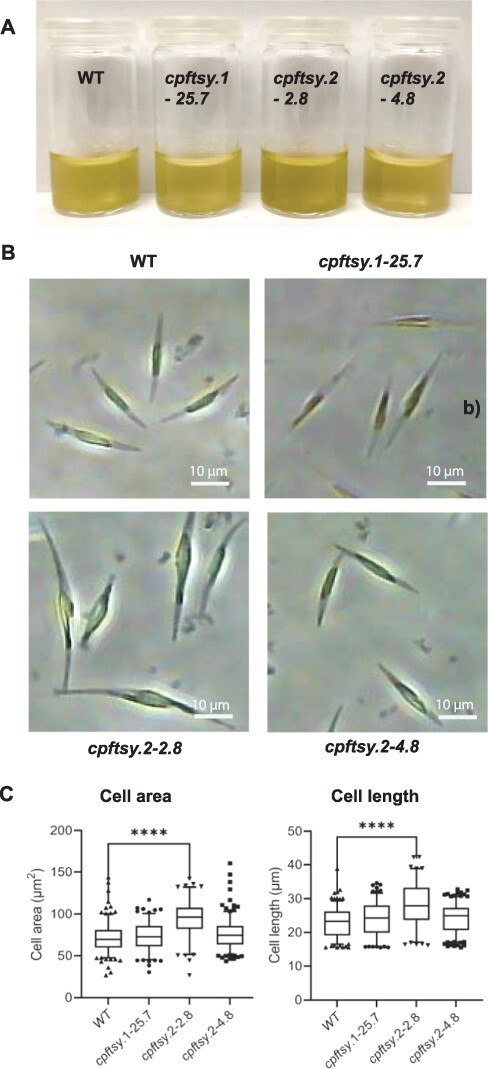
The culture color and cell size of WT and *cpftsy* lines. (A) LL-acclimated cultures up-concentrated to 15 million cells ml^−1^. (B) Representative phase-contrast images of WT and *cpftsy* mutant cells. (C) The area and length of WT and *cpftsy* mutant cells. Box and whisker plots represent the mean and 5th–95th percentile of >100 cells per group (****, *P* < 0.0001).

**Fig. 3:**
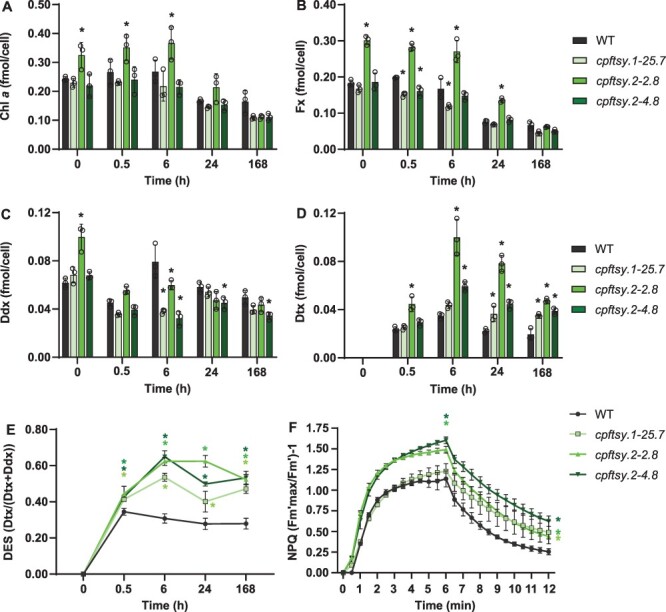
The cellular pigment concentration, DES index and NPQ of WT and *cpftsy* mutants. (A) Chl *a*, (B) Fx, (C) Ddx, (D) Dtx and (E) DES index [calculated from the values shown in (C) and (D)] as a function of 0.5, 6, 24 and 168 h of ML exposure time. The 0-h time point represents LL-acclimated samples. Black circles indicate individual data points for replicates. (F) NPQ induction (0–6 min; 470 µmol photons m^−2^ s^−1^ of blue light) and relaxation 6–12 min (8 µmol photons m^−2^ s^−1^ of blue light) of LL-acclimated cells. All results are presented as means of three biological replicates ± SD. Asterisks describe significant differences between c*pftsy* mutants and the WT as indicated by two-way ANOVA with Dunnett’s multiple comparison tests (*P* < 0.05). In (F), the results of the two-way ANOVA test are only shown for the last data point for NPQ induction (6 min) and for the last data point for NPQ relaxation (12 min) because of lack of space.

Whereas the lack of the ALB3b insertase resulted in a color change from brown to green in *P. tricornutum* because of a decline of the LHCP content, no such effects were observed in the *cpsrp54* mutants (Nymark et al. 2019, 2021). As expected, based on the assumption that CpFTSY is the receptor of CpSRP54, *cpftsy* mutant lines also showed a similar brown color as the WT, except for *cpftsy.2-2.8* appearing darker brown (**Fig. 2A**). As described in more detail later, pigment analyses of the different cell lines confirmed a higher pigment content per cell in the *cpftsy.2-2.8* line compared to the other mutants and the WT (**Fig. 3**), but the pigment ratios per chlorophyll (Chl) *a* were similar, indicating that the higher pigment content per cell was not caused by a change in the antenna size per photosystem (**Supplementary Fig. S4**). Normalized absorption spectra derived from the WT and the three *cpftsy* lines were all close to identical, confirming the observations from the pigment analyses (**Supplementary Fig. S5**). Microscopic analysis revealed that the *cpftsy.2-2.8* cells were significantly larger than the WT and the other *cpftsy* mutant lines (**Fig. 2B, C**). The same gRNA was used to create the *cpftsy.2-2.8* and *cpftsy.2-4.8* lines, but the sites of genomic integration of the vector DNA were different and might explain the peculiar phenotype of the *cpftsy.2-2.8* line. One of the integration sites of vector DNA in the *cpftsy.2-2.8* line was at position 495411 in Chr21 (OU594962.1) and was in the upstream regulatory region of Phatr3_J49177, a topoisomerase related function 4 domain–containing protein that may encode a nucleotidyltransferase. The Phatr3_J49177 protein has an unknown function but shows similarity to Cid1-type nucleotidyltransferases that have been tied to cell cycle progression in other organisms (Read et al. 2002, Olsen et al. 2006, Rissland et al. 2007). It could be speculated that the integration of vector DNA in the upstream regulatory region of the Phatr3_J49177 gene causes inappropriate expression patterns of the gene/protein, disturbs the cell cycle progression and influences the cell size of the *cpftsy.2-2.8* line. The *cpftsy.2-2.8* line also shows slower growth rates than the other *cpftsy* mutants (**Table 1**).

**Table 1:** Growth rates of WT and *cpftsy* mutant lines acclimated to LL or ML

	35 µmol s^−1^ m^−2^	200 µmol s^−1^ m^−2^
WT	1.42 ± 0.08	1.95 ± 0.02
*cpftsy.1-25.7*	0.87 ± 0.03	1.51 ± 0.05
*cpftsy.2-2.8*	0.64 ± 0.06	0.67 ± 0.03
*cpftsy.2-4.8*	0.96 ± 0.10	1.27 ± 0.05

The maximum cell divisions per day during the exponential phase were calculated from three biological replicates of WT and *cpftsy* mutant lines acclimated to LL (35 µmol photons m^−2^ s^−1^) or ML (200 µmol photons m^−2^ s^−1^). Values are presented as mean ± SD.

### Strong phenotypic resemblance between *cpftsy* and *cpsrp54* mutants

**Fig. 4:**
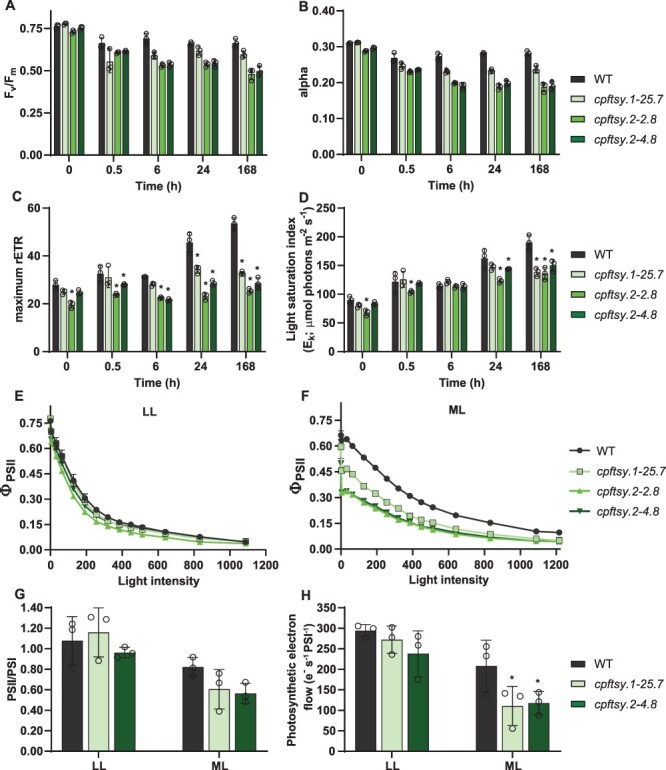
Photophysiological responses of *cpftsy* mutants and WT. (A) The photosynthetic (PSII) efficiency (*F*_v_/*F*_m_), (B) the maximum light utilization coefficient (alpha), (C) the photosynthetic capacity (rETR_max_) and (D) the light saturation index (*E*_k_) as a function of 0.5, 6, 24 and 168 h of ML exposure time in the WT and *cpftsy* mutants. The 0-h time point represents LL-acclimated samples. The quantum yield of PSII (Φ_PSII_) as a function of stepwise increasing irradiances at intervals of 30 s in (E) LL-acclimated and (F) ML-acclimated (168 h) cells. (G) In vivo assessment of functional PSII/PSI RC ratios and (H) photosynthetic electron flow in LL- and ML-acclimated (168 h) cultures of WT, *cpftsy.1-25.7* and *cpftsy.2-4.8* lines. All results are presented as means of three biological replicates ± SD. Black circles indicate individual data points for replicates. Asterisks describe significant differences between c*pftsy* mutants and the WT as indicated by two-way ANOVA with Dunnett’s multiple comparison tests (*P* < 0.05).

**Fig. 5:**
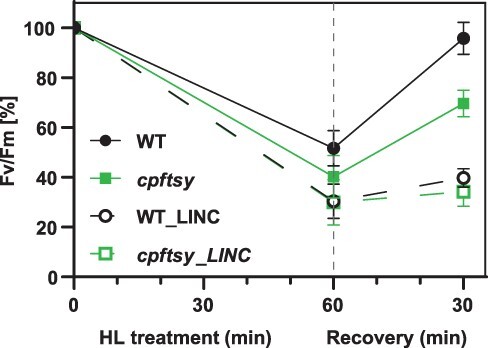
Responses of photosynthetic efficiency to HL treatment with and without the presence of LINC. LL-acclimated *cpftsy* and WT cultures were exposed to HL (solid lines) or HL + LINC (dotted line) for 60 min before relaxation in very dim light for 30 min. The responses of the three independent *cpftsy* mutants to the treatment were highly similar, and the graph represents the means of three biological replicates ± SD from each of the three lines (*n* = 9). Three biological replicates were also used for the WT.

We have previously reported that in contrast to plants and green algae, the lack of CpSRP54 proteins does not have a negative impact on the LHCP and pigment content in diatoms but causes a light-sensitive phenotype (Nymark et al. 2021). When exposing LL-acclimated *cpsrp54* cells to medium light (ML), *cpsrp54* mutants increased the levels of the photoprotective carotenoid diatoxanthin (Dtx) and displayed significantly lower photosynthetic electron transport rates, and the fraction of functional PSII reaction centers (RCs) per functional PSI RC strongly declined compared to the WT (Nymark et al. 2021). The photoprotective mechanism non-photochemical quenching (NPQ) of chlorophyll fluorescence, enabling cells to dissipate excessively absorbed energy harmlessly as heat, was also induced to a higher level when CpSRP54 was lacking. In addition, experiments with HL intensities in combination with an inhibitor of chloroplast protein synthesis showed that the repair of PSII was inefficient in *cpsrp54* mutants (Nymark et al. 2021). When tested in the same conditions, *cpftsy* mutants displayed similar phenotypes as *cpsrp54* lines, revealing a strong phenotypic resemblance between the two genotypes (**Figs. 3**–**5**), in line with the assumption that CpFTSY is involved in the same pathway as CpSRP54. The results of these measurements are described in more detail and discussed in the following sections.

### *cpftsy* mutants have a light-sensitive phenotype

As for the *cpsrp54* mutant lines, quantitative measurements of pigment content in the *cpftsy* lines revealed comparable levels of Chl *a* and Fx per cell as the WT, except for *cpftsy.2-2.8* as mentioned previously (**Fig. 3A, B**), both in LL-acclimated cells and after different exposure times to ML. The results are in support of the model presented by Nymark et al. (2021), where CpSRP54 and CpFTSY are hypothesized not to take part in the post-translational insertion of LHCPs into thylakoid membranes in diatoms. Consistent with its appearance, the *cpftsy.2-2.8* line displayed generally higher cellular concentrations of Chl *a* and Fx in LL and during acclimation to ML (0.5–24 h; **Fig. 3A, B**). The content of the xanthophyll cycle pigment diadinoxanthin (Ddx) was also higher in LL-acclimated *cpftsy.2-2.8* cells, whereas no differences in pigment content were detected between the two other mutant lines (*cpftsy.1-25.7* and *cpftsy.2-4.8*) and the WT under these conditions (**Fig. 3C**). Exposure to increased light intensities induces photoprotective mechanisms, aiming to avoid damage to the photosynthetic apparatus (Li et al. 2009, Nymark et al. 2009, Depauw et al. 2012). This involves the de-epoxidation of Ddx to Dtx, which is one of the components necessary for the induction of the photoprotective mechanism NPQ (Goss and Lepetit 2015). The Ddx and Dtx contents were similar in *cpftsy* mutants and the WT after short-term exposure (0.5 h) to ML, but prolonged exposure to ML resulted in more pronounced differences (**Fig. 3C, D**). At the later time points, a larger fraction of Ddx was converted into Dtx in the mutants compared to the WT as indicated by the higher de-epoxidation state (DES) index [Dtx/(Dtx + Ddx)] (**Fig. 3E**). NPQ measurements performed on LL-acclimated cells also indicated a more light-stressed phenotype with two out of the three mutants reaching higher NPQ values than the WT during 6 min of high-intensity (470 µmol photons m^−2^ s^−1^) blue-light exposure and all three mutants remaining in a more photoprotective state after 6 min of relaxation in very dim light (**Fig. 3F**).

The photophysiological status of the cells was assessed using Chl *a* variable fluorescence for calculations of the photosynthetic (PSII) efficiency (maximum quantum yield, *F*_v_/*F*_m_), the quantum yield of PSII (Φ_PSII_), the maximum light utilization coefficient (the slope of the photosynthesis versus irradiance curves, alpha), the photosynthetic capacity (maximum relative electron transport rate, rETR_max_) and the light saturation index (*E*_k_ = rETR_max_/alpha) after the shift from LL to ML (**Fig. 4A****–****D**). *E*_k_, alpha and rETR_max_ were derived from rapid light curves. The measurements showed that all three *cpftsy* mutants behaved similarly as a response to the different light treatments, indicating that the differences in photosynthetic performance between mutants and the WT are the effects of lacking CpFTSY and that they are not side effects caused by unintended disturbance of other genetic elements. The photosynthetic performance of LL-acclimated *cpftsy* cells showed a weak negative trend compared to WT cells, whereas prolonged exposure to ML revealed that the photosynthetic apparatus of the *cpftsy* mutants did not function optimally at increased light pressures. With only very few exceptions, the values for all the above-mentioned parameters were lower in mutants compared to the WT during prolonged ML exposure (**Fig. 4A****–****D, 4F**). The significant drop in Φ_PSII_ in ML-acclimated cells already at very LL intensities (**Fig. 4F**) and the strongly reduced photosynthetic capacity (rETR_max_; **Fig. 4C**) of the *cpftsy* mutants were the most pronounced features. A similar reduction of the efficiency of photosynthetic electron transport was also detected in *A. thaliana cpftsy* mutants (Tzvetkova-Chevolleau et al. 2007). As also reported for the diatom *cpsrp54* mutants, the rETR_max_ in the *cpftsy* lines remained close to LL levels even after a week in ML and did not show the expected strong increase in photosynthetic capacity after acclimation of the photosynthetic apparatus to HL intensities (Nymark et al. 2009, 2021). The photophysiological characterization of the *cpftsy* mutants was completed by measuring the stoichiometry of functional RCs and the total photosynthetic electron flow in two of the mutant lines in LL and ML. These parameters were determined using the electrochromic shift (ECS), a shift of the absorption properties of membrane-embedded photosynthetic pigments that respond to the building of a *trans*-thylakoid membrane potential during photosynthesis (Witt 1979). The analyses showed that the photosynthetic electron flow in *cpftsy* mutants was ∼50% lower than that in WT cells when acclimated to ML (**Fig. 4H**), confirming that photoinhibition occurred to a larger extent in mutants lacking CpFTSY. The same phenomenon was reported for *cpsrp54* mutants (Nymark et al. 2021). For the *cpsrp54* mutants, we suggested that the explanation for the poorer photosynthetic performance of these mutants was a strong decline in the fraction of functional PSII/PSI in ML caused by inefficient repair of PSII (Nymark et al. 2021). The amount of functional PSII left in the *cpsrp54* mutants upon exposure to increased light intensities was limiting for photosynthetic activity in these lines. The assessment of functional PSII/PSI RC ratios in ML-acclimated cells for *cpftsy* revealed the same trend as for *cpsrp54*, but the differences between *cpftsy* mutants and the WT were not statistically significant (**Fig. 4G**). Consistent with the slightly reduced photosynthetic activity measured in LL-acclimated *cpftsy* mutant cells and the clear negative effects observed under ML (**Fig. 4**), the cell division rate was lowered in the *cpftsy* mutants compared to the WT (**Table 1**). Reduced photosynthetic performances in both LL and ML were confirmed by measuring the photosynthetic efficiency during growth using an AquaPen (Photon Systems Instruments, Brno, Czech Republic; **Supplementary Table S2**).

To further investigate the reason for the susceptibility of the *cpftsy* mutants to increased light intensities, we measured changes in the photosynthetic efficiency (*F*_v_/*F*_m_) after exposing LL-acclimated cells to 1 h of HL intensities (1,000 µmol photons m^−2^ s^−1^) with and without the addition of lincomycin (LINC), an inhibitor of protein synthesis in the chloroplast. The PSII repair mechanism depends largely on the *de novo* synthesis of proteins, particularly the PSII core protein D1 is known to be prone to photodamage and has a high turnover rate (Aro et al. 1993, 2005, Jansen et al. 1996, Rokka et al. 2005). In diatoms, the PSII core protein D2 has been shown to have a similarly high turnover rate as D1 (Wu et al. 2011, 2012). A frequent and efficient replacement of photodamaged proteins is vital for securing PSII functionality during periods of HL (Aro et al. 1993). *F*_v_/*F*_m_ values measured after 1 h in HL will be influenced by both the thermal dissipation of absorbed energy (qE) and the photodamage of PSII. The degree of photodamage will depend on both the rate of photodamage and the rate of PSII repair. Blocking the ability to replace photodamaged proteins by the addition of LINC to the algae cultures enables separation between photodamage and repair, whereas the 30-min recovery period in very dim light after HL exposure is expected to eliminate the contribution from qE. HL exposure of cultures, where the ability to perform PSII repair was inhibited, caused an identical and severe decline in *F*_v_/*F*_m_ to 30% of the initial value in both the WT and mutants after 1 h of HL treatment, indicating that there is no difference in the degree of photodamage between *cpftsy* mutants and the WT (**Fig. 5**). HL exposure without the presence of LINC caused a decline in *F*_v_/*F*_m_ to 40% and 52% of the initial value in mutants and the WT, respectively (**Fig. 5**). The WT recovered almost completely (96% of the initial value) during the 30-min period of very dim light treatment, whereas the mutants showed a significantly lower recovery of only ∼70% of the initial value. The moderately lower *F*_v_/*F*_m_ in *cpftsy* compared to the WT after HL treatment implies a less-efficient repair of PSII in the mutants, but the lower ability to recover during the 30-min period in very dim light after HL treatment suggests that also other long-lived fluorescence quenching mechanisms might be affected to a different degree in *cpftsy* mutants compared to the WT. In contrast to the moderate effect on PSII repair observed in diatom *cpftsy* mutants, high-intensity light treatments of *A. thaliana cpftsy* mutants revealed a severely reduced PSII repair mechanism and no recovery of the D1 protein level nor *F*_v_/*F*_m_ even after 90 min of recovery in very dim light (Walter et al. 2015). The inefficient PSII repair mechanism in *A. thaliana cpftsy* mutants was explained by an impaired binding of translating ribosomes to the thylakoid membrane when CpFTSY was absent (Walter et al. 2015). *Phaeodactylum tricornutum* CpFTSY proteins might also be important for the association between ribosomes and thylakoid membranes, considering the presence of N-terminal amphipathic structures known from *A. thaliana* and *E. coli* to be involved in membrane binding. Despite the phenotype of the *P. tricornutum cpftsy* mutants being less severe than that reported in the literature for the corresponding mutants in *A. thaliana*, the results described above do indicate a role for CpFTSY in PSII repair and the replacement of photodamaged proteins also in diatoms.

### Effects of loss of CpFTSY on the amount of chloroplast proteins

Proteomic analyses revealed moderately lower levels of various chloroplast-encoded subunits of the photosynthetic complexes in both plant and diatom *cpsrp54* mutants (Rutschow et al. 2008, Nymark et al. 2021). In plants, a range of different LHCPs were also found to be negatively affected by the lack of CpSRP54 (Rutschow et al. 2008). Proteomic data are not available for plant or green algae *cpftsy* mutants, but protein analyses of selected subunits of the photosynthetic apparatus revealed a downregulation of LHCPs and a range of chloroplast-encoded photosynthetic thylakoid membrane proteins (Asakura et al. 2004, Tzvetkova-Chevolleau et al. 2007, Kirst et al. 2012a). However, the downregulation of chloroplast-encoded subunits of photosynthetic complexes in green algae was not interpreted as *cpftsy* having a role in the co-translational CpSRP pathway, but rather to be an indirect consequence of overall lower thylakoid membrane abundance in the chloroplast of the *tla2* mutant (Kirst et al. 2012a). To investigate the effect of the lack of CpFTSY in diatoms on chloroplast proteins, we performed a proteomic analysis on the WT and two of the *cpftsy* lines (*cpftsy.1-25.7* and *cpftsy.2-4.8*) using five biological replicates from each line. We chose to use samples acclimated to ML for the proteomic analyses since the photophysiological responses were clearly different in mutants and the WT under these light conditions (**Fig. 4**). Proteins predicted to be localized to the chloroplast that was significantly up- or downregulated [false discovery rate (FDR) < 0.05] in the same direction in both *cpftsy* lines, showing log_2_ ratios ≥±0.5 for at least one of the mutant lines and where at least two peptides were detected, are included in **Table 2**. The most interesting findings will be discussed in the following section.

**Table 2:** Proteomics data for *cpftsy* lines compared to the WT after acclimation to ML

Protein ID	Protein description	Function (predicted)	cpftsy.1-25.7/WT (log_2_ ratio)	cpftsy.2-4.8/WT (log_2_ ratio)	Location (predicted)	Nucleus (N)/Chloroplast (Cp)-encoded	No. of unique peptides	No. of peptides	Score Sequest HT
A0T0B7	PetD	Cyt*b*_6_*f*—electron transport	**−0.39**	**−0.53**	TM	Cp	3	3	33.1
A0T0L2	PsaC	PSI—electron transport	**−1.35**	**−1.96**	TM	Cp	4	4	176.1
B7FXG3	NADH dehydrogenase	Electron transport	1.10	1.14	TM	N	9	9	47.5
B7GBK7	LHCF5	Light harvesting	3.61	3.12	TM	N	1	4	261.1
B7G6Y1	LHCF8	Light harvesting	1.29	1.15	TM	N	7	10	560.0
B7G5B6	LHCF10	Light harvesting	1.39	1.52	TM	N	3	5	315.9
B7GBK6	LHCF11	Light harvesting	1.69	1.29	TM	N	1	4	446.6
B7G8E5	LHCQ2	Light harvesting	1.16	1.11	TM	N	5	5	174.8
B7G4U8	LHCR6	Photoprotection	0.84	0.95	TM	N	7	7	84.8
B7FYL0	LHCX1	Photoprotection	1.17	1.49	TM	N	4	4	317.7
B7FNX5	LCYB	Pigment synthesis	0.76	0.86	Cp	N	2	2	10.9
B7G0M8	COBA	Pigment synthesis	0.97	1.07	S	N	2	2	2.7
B7GDU9	PPO	Pigment synthesis	1.07	1.08	S	N	7	7	28.4
B5Y3F4	CHLH	Pigment synthesis	1.11	1.49	S	N	40	40	439.5
B7FTQ8	CHLM	Pigment synthesis	0.64	0.80	S	N	10	10	133.1
B7FY80	POR	Pigment synthesis	1.03	0.85	S	N	8	8	51.2
B7FW30	CBR	Pigment degradation	2.12	1.64	TM	N	7	7	78.7
B7GD45	RCCR	Pigment degradation	1.45	1.93	S	N	2	2	13.6
B7FT10	ALB3a	TM insertase activity	1.24	1.86	TM	N	6	6	99.1
B5Y591	HCF101-homolog	Fe–S cluster biosynthesis	0.72	0.77	S	N	4	4	9.3
B7FVZ9 ^a^	CPSUFE	Fe–S cluster biosynthesis	0.48	0.71	S	N	1	1	18.6
B7FWZ2	Fe–S assembly-like protein	Fe–S cluster biosynthesis	1.36	2.36	Cp	N	7	7	26.4
B7G7J9	APE1-homolog	Photoacclimation	1.16	1.58	TM	N	5	5	52.5
B5Y3Z3	ABC1K6	Stress responses	0.71	1.16	PG	N	7	7	19.5
B7FY63	ABC1K7/8-like protein	Stress responses	1.27	1.55	PG	N	1	2	12.9
B7G0A5	FBN6-like	Stress responses	1.16	1.21	PG	N	3	3	15.7
B7FQN7	FBN17	Stress responses	0.83	1.25	PG	N	4	4	16.7
B7FW72	PP2C	Serine/threonine phosphatase activity	1.21	1.03	Cp	N	2	2	6.4
B7G1T3	CDC48	Chloroplast protein degradation	1.19	1.62	CpM	N	26	27	362.9
B7FZ42	ACSL	Fatty acid metabolism	1.15	1.55	C	N	6	6	18.2
B5Y5J4	MPBQ/MSBQ transferase	Tocopherol synthesis	1.07	1.41	CpM	N	4	4	32.4
B5Y3Q7	Methyltransferase	Methyltransferase activity	1.24	1.80	Cp	N	3	3	7.5
B7FR53	TPP	Signal peptide processing	0.76	1.03	L	N	4	4	35.9
B7GEF3	TIC110	Chloroplast protein import	0.89	0.82	CpM	N	32	32	422.4
B7G1F1	TIC22	Chloroplast protein import	1.34	1.33	CpM	N	8	8	64.9
B7FUD8	Tic62-NAD(P)-related group II protein	Unknown	0.80	0.65	Cp	N	14	14	266.7
B5Y5F0	PRK	Carbon fixation	0.77	1.17	S	N	10	10	213.3
B7FXP8	CA2	Carbon-concentrating mechanism	1.20	1.91	Cp	N	7	11	538.4
B7FR28	CA	Carbon-concentrating mechanism	0.92	1.13	L	N	15	15	194.8
A0T0H7	DnaK	Chaperone	1.83	2.10	S	Cp	47	47	1892.6
B7GDN1	HSP90	Chaperone	1.71	2.28	S	N	34	35	754.0
B7GEF7	HSP90	Chaperone	0.94	1.16	S	N	33	33	626.6
B5Y3P1	DNAJ	Co-chaperone	1.08	1.55	S	N	20	20	261.3
B5Y5I5	CLPB	Chaperone	1.40	2.16	S	N	34	36	434.2
B7S404	Trigger factor	Chaperone	0.45	0.71	S	N	32	32	557.0
B7FX39	Cyclophilin-type peptidyl-prolyl *cis*–*trans* isomerase	Protein folding	**−1.15**	**−1.12**	Cp	N	9	11	451.8
B7FPG9	Oligopeptidase A	Hydrolysis of peptide bond	0.80	1.42	Cp	N	21	21	320.9
B7FQC3	RPS21	Translation	**−0.96**	**−0.88**	CpR	N	4	4	144.0
A0T0C2	Rpl11	Translation	**−0.48**	**−0.38**	CpR	Cp	9	9	270.7
A0T0J0	Rpl24	Translation	**−0.94**	**−0.70**	CpR	Cp	7	7	147.5
B7G0B8	Peptide chain release factor	Translational termination	1.05	1.59	Cp	N	3	3	13.2
B5Y3B2	Myo-inositol 2-dehydrogenase	Metabolism	1.41	2.83	Cp	N	17	17	220.6
B7FSN9	Anion-transporting ATPase	Unknown	1.14	2.09	CpM	N	15	15	108.7
B7FQF2	RNA recognition motif protein	Unknown	0.51	1.27	Cp	N	11	11	138.0
B7G4H4	EMP70-family protein	Unknown	0.99	0.96	CpM	N	3	3	24.7
B7G849	Bacterial PH domain protein	Unknown	0.75	1.28	Cp	N	3	3	15.2
B7G128	DUF1118-family protein	Unknown	0.78	0.97	Cp	N	4	4	188.6
B7G2G3	Unknown	Unknown	0.77	2.05	Cp	N	9	9	239.2
B7FW47	Unknown	Unknown	0.93	0.57	Cp	N	16	16	88.4
B7G249	Unknown	Unknown	1.07	1.09	Cp	N	11	11	258.7
B7FQJ5	Unknown	Unknown	0.57	1.24	Cp	N	6	6	80.5
B7FY71	Unknown	Unknown	1.21	1.02	Cp	N	5	5	57.4
B7GC65	Unknown	Unknown	0.49	0.60	Cp	N	7	7	42.5
B7G5H8	Unknown	Unknown	1.33	1.87	Cp	N	8	8	68.6
B7FW98	Unknown	Unknown	2.82	5.41	Cp	N	11	11	128.9

Psa: PSI protein; Pet: Cyt*b*_6_*f* protein; LHC: light-harvesting complex; LHCF: major Fx–Chl *a/c* proteins; LHCR: red algal–like proteins; LHCX: LI818-like (stress-related) proteins; LCYB: lycopene β-cyclase; COBA: uroporphyrinogen-III C-methyltransferase; PPO: protoporphynogen oxidase; CHLH: Protoporphyrin IX magnesium chelatase, subunit H; CHLM: Mg-protoporphyrin IX methyl transferase; POR: NADPH-protochlorophyllide oxidoreductase; CBR: Chl reductase; RCCR: red Chl catabolite reductase; ALB3a: albino 3a insertase; HCF101: high-Chl-fluorescence 101; CPSUFE: SufE- and BolA-domain protein; APE1: acclimation of photosynthesis to environment 1; ABC1K: activity of bc1 complex kinase; FBN: fibrillin; PP2C: protein phosphatase 2C; CDC48: cell division cycle protein 48; ACSL: long-chain acyl-CoA synthetase; MPBQ/MSBQ: 2-methyl-6-phytyl-1,4-hydroquinone/2-methyl-6-solanyl-1,4-benzoquinone; TPP: thylakoidal processing peptidase; TIC: translocator of the inner chloroplast envelope membrane; PRK: phosphoribulokinase; CA: carbonic anhydrase; HSP: heat shock protein; CLPB: caseinolytic proteases of subfamily B; RPS: 30S ribosomal protein; RPL: 50S ribosomal protein; EMP70: endomembrane protein 70; N: nucleus; Cp: chloroplast; TM: thylakoid membrane; CpM: chloroplast membrane; S: stroma; PG: plastoglobuli; L: lumen; CpR: chloroplast ribosome.

Proteins encoded in the chloroplast genome or predicted to contain chloroplast transit peptide sequences that were significantly regulated (FDR < 0.05) in the same direction in both *cpftsy.1-25.7* and *cpftsy.2-4.8* lines compared to WT in ML-acclimated cells showing log_2_ ratios ≥±0.5 for at least one of the mutant lines and where at least two peptides were detected. Ratios were calculated based on results from five biological replicates for each line. Downregulated proteins are marked in bold.

a Low-molecular-weight protein included despite the detection of only one peptide.

### LHCPs were unaffected or upregulated in *P. tricornutum cpftsy* mutants, indicating that CpFTSY is unlikely to play a role in the targeting of LHCPs to thylakoid membranes in diatoms

*Phaeodactylum tricornutum* contains >40 proteins belonging to the LHCP family, and these proteins are divided into several subfamilies (Nymark et al. 2013, Kumazawa et al. 2022). Four proteins of the LHCF family (the major Fx–Chl *a/c* binding proteins) and a protein of the PSI-associated LHCQ family were found to be upregulated in *cpftsy* under ML conditions (**Table 2**). The increased LHCP levels were accompanied by a moderate upregulation of four enzymes (PPO, CHLH, CHLM and POR) in the multistep Chl *a* biosynthetic pathway and LCYB involved in the synthesis of carotenoids (**Table 2**). These responses could imply a larger antenna in *cpftsy* mutants than in the WT, but this implication is not supported by the measurements of Chl *a* and Fx content per cell, showing no significant differences between mutants and the WT in ML conditions (**Fig. 3A, B**). The poorer photosynthetic performance of the mutants might instead have triggered a chloroplast-to-nucleus retrograde signal, leading to the increased expression of the above-mentioned proteins (Tanaka and Tanaka 2007, Stenbaek and Jensen 2010, Yuan et al. 2017). However, the excess accumulation of Chl and Chl intermediates that are not assembled into antenna complexes is phototoxic and needs to be avoided (Tanaka and Tanaka 2007). Diatom homologs of two enzymes known from plants to be involved in Chl degradation through the pheophorbide *a* oxygenase (PAO) pathway, a Chl reductase (CBR) and a red Chl catabolite reductase (RCCR) showed a ∼3–4-fold upregulation and might counteract a potential build-up of excess Chls (Hörtensteiner and Kräutler 2011). In addition to the upregulation of proteins involved in light harvesting, two LHCPs with confirmed or predicted roles in photoprotection were also found to be upregulated in the *cpftsy* mutants: LHCX1, which is essential for NPQ to take place (Bailleul et al. 2010b, Buck et al. 2019), and the red algal–like LHCR6, which is known to be induced by increased light intensities (Nymark et al. 2009, Lepetit et al. 2010). The higher amounts of photoprotective LHCPs support the photophysiological responses, indicating increased light stress in *cpftsy* when exposed to ML. The above-described results for the level of LHCPs (**Table 2**) and light-harvesting pigments (**Fig. 3**) in *cpftsy* compared to the WT are clearly not consistent with a role for CpFTSY in a post-translational targeting mechanism of LHCPs in diatoms, at variance with earlier conclusions derived from plants and green algae (Asakura et al. 2004, Tzvetkova-Chevolleau et al. 2007, Kirst et al. 2012a).

### Loss of CpFTSY causes a strong decline in PsaC of PSI and triggers the induction of proteins involved in chloroplastic Fe–S cluster formation

Photosynthetic electron transport and ATP synthesis involve the four multisubunit complexes PSII, Cyt*b*_6_*f*, PSI and ATP synthase, which comprise both chloroplast and nucleus-encoded subunits (Eberhard et al. 2008). The lack of CpSRP54 in diatoms caused moderately lowered levels of several chloroplast-encoded proteins of all the above-mentioned complexes, with D2 (PsbD) of PSII being the most affected (Nymark et al. 2021). The same general downregulation of subunits of photosynthetic complexes was not found in the *cpftsy* mutants. Instead, we observed a modest downregulation of subunit IV (PetD) of the Cyt*b*_6_*f* complex and, most noticeably, a significant decline of the PsaC of PSI to only 25–39% of WT levels in the two *cpftsy* mutants (**Table 2**). PsaC was also among the downregulated PSI subunits detected in diatom *cpsrp54* mutants, but the effect of the absence of CpSRP54 on PsaC levels was much more moderate than in mutants lacking CpFTSY (Nymark et al. 2021). The protein level of PsaC has previously also been investigated in maize *cpftsy* mutants, where it was reported to be strongly downregulated (Asakura et al. 2004). PsaC is a stromal subunit of PSI and is not integrated into the thylakoid membrane (Nagao et al. 2020). The observed downregulation of PsaC in maize was interpreted as being an indirect effect of lacking other PSI subunits, but our results could indicate a more direct involvement of CpFTSY in the targeting of PsaC to the stromal side of the thylakoid membranes. The accumulation of PSI complexes without essential subunits is unexpected, based on the notion that a tight interplay exists between complex assembly and translational regulation of photosynthetic complexes (Choquet and Wollman 2002). Subunits PsaA–PsaF and PsaL of PSI were all detected through the proteomic analyses (**Supplementary Table S3**). In addition to the downregulation of PsaC, a possible downregulation also of PsaD was indicated in both *cpftsy* lines (66–70% of WT levels), but the downregulation was not statistically significant (**Supplementary Table S3**). We note, however, that previous work in *A. thaliana* (Tomizioli et al. 2014) and cyanobacteria (Mannan et al. 1991) already reported the presence of a PSI complex with altered PsaC levels in the thylakoid membranes. PsaC contains two (4 Fe–4 S) clusters that function as the terminal electron acceptors of PSI, and the outermost cluster works in the transfer of electrons from PSI to ferredoxin (Fischer et al. 1999, Busch and Hippler 2011). Thus, lower amounts of PsaC subunits would inhibit the flow of electrons through PSI and cause the low photosynthetic electron transport rate measured in the *cpftsy* mutants (**Fig. 4C, F, H**) in ML in this study and also in a study with *A. thaliana cpftsy* (Tzvetkova-Chevolleau et al. 2007). The expression of homologs of proteins known from plants to play a role in the formation of Fe–S clusters in the chloroplast was increased in the diatom *cpftsy* mutants (**Table 2**). HCF101 is universally conserved and essential for the assembly of the [4 Fe–4 S]-containing PSI in plants (Lezhneva et al. 2004), whereas the SufE- and BolA-domain protein detected in our analyses is an ortholog of the *A. thaliana* CpSufE that activates the cysteine desulfurase CpNifS for chloroplastic Fe–S cluster formation (Ye et al. 2006). Lastly, a homolog of an uncharacterized *A. thaliana* protein classified as an Fe–S cluster biosynthesis family protein displayed the strongest upregulation (∼2.5- to 5-fold) of the proteins predicted to be involved in the formation of Fe–S clusters. Durrett et al. (2006) suggested that plant *cpftsy* is involved in the activation of Fe(III) chelate reductase activity in roots through an unknown mechanism in addition to its role in the CpSRP pathway. The upregulation of proteins involved in Fe–S cluster formation is likely to be triggered in a consequence of a low PsaC content caused by inefficient targeting of this protein to the stromal side of the thylakoid membranes in *cpftsy* mutants. Another possibility would be that the lack of diatom CpFTSY might somehow have a negative effect on the Fe uptake mechanism in the mutant cells and that the lower amount of PsaC and the increased expression of Fe–S cluster formation proteins are responses to a lack of available Fe. This explanation is, however, unlikely since the intracellular Fe (Fe-to-P ratio) determined in both mutants and the WT under the experimental conditions used for the proteomic analyses was found to be well within the Fe quota exhibited for different diatoms (**Supplementary Table S4**) (Twining and Baines 2013, Gao et al. 2021). Additionally, a general lack of Fe in *P. tricornutum* cells has been reported not only to have a negative effect on the abundance of Fe-containing subunits of the photosynthetic electron transport chain but also to result in smaller cells, a strong decline in the Chl content and a low *F*_v_/*F*_m_ regardless of light intensity, which are the effects not observed in the *cpftsy* mutants (Allen et al. 2008, Roncel et al. 2016).

### Mechanisms compensating for a disrupted co-translational CpSRP pathway are induced in *cpftsy* mutants

The moderate negative effects of the lack of CpSRP54 on the amount of thylakoid membrane proteins in both plants and diatoms were explained by an increased expression of a wide range of chloroplast-localized chaperones and other proteins predicted to be involved in compensatory mechanisms, preventing the accumulation of misfolded or aggregated membrane proteins and assisting in the insertion of photosynthetic proteins into thylakoid membranes (Rutschow et al. 2008, Ries et al. 2020, Nymark et al. 2021). An upregulation of stromal chaperones and components of the thylakoid protein translocation machinery has also been reported in maize *cpftsy* mutants (Asakura et al. 2004). Trigger factor is a ribosome-associated ATP-independent chaperone found in bacteria and chloroplasts that receives emerging nascent polypeptides, thereby preventing misfolding and triggering the folding of the proteins being synthesized (Ries et al. 2020). Other co-translational acting chaperones in chloroplasts of the green lineage are HSP70 DnaK and its co-chaperones, HSP90C and CPN60 (Ries et al. 2020). Diatom homologs of trigger factor, HSP70 DnaK (chloroplast-encoded), DNAJ (DnaK co-chaperone) and two chaperones of the HSP90 family predicted to be localized to chloroplasts were found to be upregulated in *cpftsy* mutants in this study (**Table 2**). Additionally, a homolog of CLPB, which is a member of the HSP100 family known to be able to disentangle protein aggregates in conjunction with the HSP70 DnaK system (Doyle et al. 2015, Mogk et al. 2015, Mishra and Grover 2016), was present at increased levels in the *cpftsy* lines (**Table 2**). HSP70 DnaK, DNAJ and CLPB were also upregulated in diatom *cpsrp54* mutants (Nymark et al. 2021). No functional data exist for the diatom homologs of these chaperones, so additional functions or other functions described previously are of course possible. However, an increased expression of the chloroplast chaperone systems in mutants lacking either CpSRP54 or CpFTSY in both plants and diatoms supports a similar role in bypassing the non-functional co-translational CpSRP pathway for the targeting of proteins to thylakoid membranes. In addition to the upregulation of chaperones, the absence of CpFTSY caused an increase in the abundance of ALB3a in diatoms (**Table 2**) and ALB3 of the CpSRP pathway in plants (Asakura et al. 2004). We have hypothesized that ALB3a functions in the co-translational CpSRP pathway together with CpSRP54 and CpFTSY, but in contrast to the two latter proteins, ALB3a is likely essential for the insertion of photosynthetic thylakoid membrane proteins (Nymark et al. 2019), similar to ALB3 in plants (Sundberg et al. 1997) and ALB3.2 in green algae (Göhre et al. 2006). An increase of ALB3a is likely also to be a compensatory mechanism induced by the loss of CpFTSY.

### Increase in the abundance of proteins with roles in photoprotection

In addition to the photoprotective antenna proteins LHCX and LHCR6 described previously, we also detected increased amounts of other proteins possibly involved in light stress responses. The activity of bc1 complex kinase (ABC1K) 6 and ABC1K7/8-like proteins and plastid-lipid-associated protein (PAP)/fibrillin 6–like (FBN6-like) and FBN17 are members of the two most abundant families of plastoglobule proteins (Lohscheider and Bartulos 2016) and were all found to be expressed at higher levels in *cpftsy* than in the WT (**Table 2**). Plastoglobuli are lipophilic droplets attached to thylakoid membranes known from studies in plants to be involved in Chl degradation, homeostasis of plastoquinone, photo-oxidative stress tolerance, regulation of tocopherol metabolism, distribution of Fe within the chloroplast, stabilization of LHCPs in photosynthetic complexes and lipid remodeling of thylakoid membranes under light stress (Martinis et al. 2014, Manara et al. 2016, van Wijk and Kessler 2017, Pralon et al. 2020). Given the unknown role of plastoglobule proteins in diatoms, we suggest that they could be interesting targets for future studies of chloroplast metabolism and photoprotection in this group of microalgae. The stromal HSP70 DnaK in the green lineage has been reported to have several different functions besides protein folding, including a role in photoprotection and PSII maintenance during light stress (Schroda et al. 1999, 2001, Trösch et al. 2015). With HSP70 DnaK being among the strongest upregulated proteins in both the diatom *cpftsy* and *cpsrp54* proteomic datasets and both mutants showing signs of photoinhibition when exposed to ML, an elevated expression level of HSP70 DnaK might be an attempt to counteract the slower PSII repair process indicated in these mutants (**Table 2**, **Fig. 5**) (Nymark et al. 2021). Lastly, a homolog of the acclimation of photosynthesis to environment 1 (APE1) protein was expressed 2- to 3-fold higher in *cpftsy* mutants than in WT cells. APE1 is a thylakoid membrane protein conserved in photosynthetic organisms and is important for long-term HL acclimation in both plants and green algae, possibly by acting as a regulator of PSII supercomplex dynamics (Walters et al. 2003, Chazaux et al. 2020).

## Conclusion

**Fig. 6:**
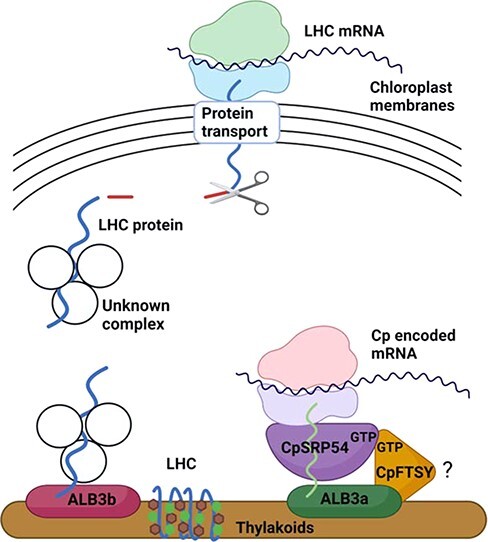
A schematic overview of the proposed role of members of the CpSRP pathway in diatoms. Nucleus-encoded LHCPs are transported across the four chloroplast membranes and guided to the ALB3b insertase through an unknown mechanism before being inserted into the thylakoid membrane (left side). A selection of chloroplast-encoded subunits of photosynthetic complexes are co-translationally inserted into thylakoid membranes assisted by the CpSRP pathway members CpSRP54, ALB3a and possibly also CpFTSY (right side). The question mark next to the CpFTSY protein indicates that the role of CpFTSY in the co-translational CpSRP pathway is uncertain. Created with BioRender.com.

The characterization of KO mutants of CpFTSY in diatoms, plants and green algae reveals that although there are similarities between the *cpftsy* mutants (**Table 3**), the severity of the phenotypes and the degree of dependence of CpFTSY for the targeting of chloroplast proteins to thylakoid membranes differ between the different groups of organisms. Whereas CpFTSY of the green lineage plays a major role in the post-translational CpSRP pathway, no evidence for such a function could be found by the investigation of the diatom *cpftsy* mutants. This finding supports our previous hypotheses that CpSRP54 and CpFTSY of the CpSRP pathway have not evolved to function post-translationally in diatoms and that the pathway for guiding LHCPs from chloroplast membranes to the ALB3b insertase in the thylakoid membranes is still unknown in this group of microalgae (**Fig. 6**). Although the proteomics data revealed only one chloroplast-encoded thylakoid membrane protein being negatively affected by the absence of diatom CpFTSY, phenotypic similarities between diatom *cpsrp54* and *cpftsy* mutants, as well as between plants and diatoms lacking CpFTSY (**Table 3**), could still suggest a role in the co-translational CpSRP pathway for the diatom CpFTSY protein. The proteomic dataset was dominated by an upregulation of a wide range of proteins that attempt to counteract the effects of a non-functional CpSRP pathway, but the light sensitivity of the *cpftsy* mutants indicated that the presence of CpFTSY is necessary for the optimal function of the photosynthetic apparatus under light stress conditions and that other mechanisms cannot fully compensate for the absence of CpFTSY under such conditions.

**Table 3:** An overview of the effects of loss of CpFTSY in the diatom *P. tricornutum*, the plants *A. thaliana* and Maize and the green algae *C. reinhardtii*

Effects of loss of CpFTSY	*P. tricornutum*	*A. thaliana*	Maize (*Zea mays*)	*C. reinhardtii*	References
Color	NC	↓	↓	↓	Present paper, Asakura et al. (2004), Durrett et al. (2006), Tzvetkova-Chevolleau et al. (2007), Kirst et al. (2012a)
LHC proteins	NC/↑	↓	↓	↓	Present paper, Asakura et al. (2004), Tzvetkova-Chevolleau et al. (2007), Kirst et al. (2012a)
Proteins of photosynthetic complexes	NC/↓	↓	↓	↓	Present paper, Asakura et al. (2004), Tzvetkova-Chevolleau et al. (2007), Kirst et al. (2012a)
Light-harvesting pigments	NC	↓	↓	↓	Present paper, Asakura et al. (2004), Tzvetkova-Chevolleau et al. (2007), Kirst et al. (2012a)
Photoprotective pigments during light stress	↑	↑	n/a	n/a	Present paper, Tzvetkova-Chevolleau et al. (2007)
Chaperones (compensating mechanisms)	↑	n/a	↑	n/a	Present paper, Asakura et al. (2004)
Growth at LL intensities	↓	↓	n/a	n/a	Present paper, Durrett et al. (2006)
Growth at HL intensities	↓	↓	seedling lethal	↓	Present paper, Asakura et al. (2004), Tzvetkova-Chevolleau et al. (2007), Kirst et al. (2012a)
Photosynthetic performance in LL intensities	NC	NC	n/a	NC	Present paper, Walter et al. (2015)
Photosynthetic performance at HL intensities	↓	↓	n/a	↑	Present paper, Tzvetkova-Chevolleau et al. (2007), Kirst et al. (2012a), Walter et al. (2015)
PSII repair	↓	↓	n/a	n/a	Present paper, Walter et al. (2015)

NC = no change; ↓ = downregulated compared to the WT; ↑ = upregulated compared to the WT; n/a = not assessed.

## Materials and Methods

### Modeling of the CpFTSY and CpSRP54 heterodimer

A 3D structural model of the diatom CpFTSY was generated based on the AlphaFold 3D prediction model of the homologous protein in *A. thaliana* (accession: AF-O80842-F1) (Baek et al. 2021, Jumper et al. 2021). The AlphaFold model of *A. thaliana* CpFTSY was used as a template for 3D model prediction of the *P. tricornutum* CpFTSY using the SWISS-MODEL database. To model the heterodimer of PtCpFTSY and PtCpSRP54, the *A. thaliana* CpFTSY–CpSRP54 dimer (Protein Data Bank ID: 5L3R) (Wild et al. 2016) and the Swiss-PdbViewer version 4.10 were used (Waterhouse et al. 2018). The PtCpSRP54 model was created using the SWISS-MODEL database prediction tool.

### *P. tricornutum cpftsy* KO mutants

*Phaeodactylum tricornutum* cultures derived from the sequenced clone Pt1 8.6 (Bowler et al. 2008) were subjected to CRISPR/Cas9-mediated mutagenesis to create *cpftsy* KO lines. All steps necessary to achieve and confirm gene-editing events in the *CpFTSY* gene (Phatr2_14412) and to isolate cells with indels causing out-of-frame mutations were performed as described previously (Nymark et al. 2016, 2017). *CpFTSY*-specific oligonucleotides used for the creation of the adapters inserted into the single-guide RNA (sgRNA) cassette of the CRISPR/Cas9 vector and primers used for screening to identify cells with mutations are presented in **Supplementary Table S5**.

### Extraction of DNA from *P. tricornutum cpftsy* KO mutants and nanopore sequencing of whole-genome libraries

*Phaeodactylum tricornutum cpftsy* lines (*cpftsy.1-25.7*, *cpftsy.2-2.8* and *cpftsy.2-4.8*) were grown to a density of approximately 1.2–1.4 × 10^6^ cells ml^−1^, and 5 × 10^8^ cells were pelleted by centrifugation for 15 min at 4000 *g* at 4°C and stored at −80°C until further processing. The cells were resuspended in a 1-ml lysis buffer (200 mM Tris HCl pH 8, 250 mM NaCl, 25 mM EGTA, 0.5% w/v SDS) and subjected to 10 freeze/thaw cycles using liquid nitrogen and a 65°C water bath. Proteinase K was added to a final concentration of 0.2 mg ml^−1^, and the samples were incubated for 60 min at 50°C, with gentle mixing by inverting the tubes every 15 min. RNase A was added to a final concentration of 0.05 mg ml^−1^, and the samples were incubated for 30 min at 37°C, with gentle mixing by inverting the tubes every 5 min. One volume of phenol:chloroform:isoamyl alcohol 25:24:1 was added, and the samples were mixed gently by inversion before being centrifuged for 5 min at 6,000 *g*. The aqueous phase was transferred to a new tube, one volume of chloroform:isoamyl alcohol 24:1 was added and the samples were mixed gently by inversion before being centrifuged for 5 min at 6,000 *g*. The aqueous phase was transferred to a new tube, 1/10 volume of sodium acetate (3 M, pH 5.2) and two volumes of ice-cold 100% ethanol were added and the samples were mixed gently by inversion. The samples were centrifuged for 5 min at 16,000 *g*, and the pellet was washed with 500 µl of 70% ethanol. The samples were centrifuged and washed once more, and the pellet was dried at room temperature and resuspended overnight at 4°C in 100 µl Tris HCl (10 mM, pH 8). DNA quality was assessed using NanoDrop One (Thermo Fisher Scientific, Waltham, MA, USA), DNA concentration was measured using a Qubit fluorometer (Thermo Fisher Scientific) and DNA integrity was visually assessed by agarose gel electrophoresis. DNA extracts were stored at −20°C until further processing. DNA libraries for nanopore sequencing were prepared from 1 µg DNA using the Native Barcoding Expansion 1-12 in conjunction with the Ligation Sequencing Kit (Oxford Nanopore Technologies, Oxford, United Kingdom) and the NEBNext Companion Module for Oxford Nanopore Technologies Ligation Sequencing (New England Biolabs, Ipswich, MA, USA) according to the manufacturer’s instructions in the Native barcoding genomic DNA protocol (version: NBE_9065_v109_revAK_14Aug2019). Libraries were kept at 4°C until being loaded on a SpotON R9.4.1 flow cell (Oxford Nanopore Technologies) and sequenced using a MinION MK1B device. Data acquisition and real-time basecalling were carried out using the MinKNOW software (Oxford Nanopore Technologies). Fastq files from the MinION runs were processed, and a BLAST database was made using a standalone server (NCBI blast-2.6.0+). The database was screened for vector integrations of the diaCas9 vector and the antibiotic selection vector pAF6 containing the *Sh ble* gene that confers zeocin resistance. MinION sequences with vector integrations were extracted, and assemblies were made with the Canu 2.2 assembler (Koren et al. 2017). Chromosomal DNA integration was identified through BLAST searches using the latest *P. tricornutum* genome assemblies (Giguere et al. 2022).

### Growth and experimental light conditions

*Phaeodactylum tricornutum* WT cells and *cpftsy* lines (*cpftsy.1-25.7*, *cpftsy.2-2.8* and *cpftsy.2-4.8*) were cultured as described previously (Nymark et al. 2009). Continuous cool white fluorescent light was used for experiments performed in LL (∼35 µmol photons m^−2^ s^−1^) and ML (∼200 µmol photons m^−2^ s^−1^) conditions. HL (∼1,000 µmol photons m^−2^ s^−1^) was provided by a full-spectrum LED lamp (5,500 K). All experiments were performed at 15°C and included three biological replicates for each line unless otherwise stated.

### Growth rates

Flow cytometry was used for the estimation of growth rates in batch cultures of WT and *cpftsy* KO lines acclimated to LL or ML as described previously using a NovoCyte™ flow cytometer (ACEA Biosciences, Santa Clara, CA, USA) (Nymark et al. 2016). Cells were kept in the exponential growth phase for at least 2 weeks before performing the experiments. Each culture was diluted to 100,000 cells ml^−1^ before starting the growth rate experiments. The average maximum cell division rates per day for WT and mutant lines were calculated by using the mean of the growth rates from the three biological replicates per line during the exponential phase. *F*_v_/*F*_m_ was monitored during the growth rate experiment using an AquaPen (Photon Systems Instruments, Czech Republic).

### Absorption spectra

Absorption spectra (400–750 nm) were measured with a Duetta spectrofluorometer (Horiba, Kyoto, Japan) with a resolution of 1 nm. Spectra were normalized to the blue absorption peak. Data are representative of three biological replicates from each cell line.

### Cell size measurements

WT and *cpftsy* mutant lines were imaged in 96-well clear plastic plates. Phase-contrast images were taken on a Nikon Eclipse TS100 microscope using the 20×, 0.4 NA Ph1 objective. For the quantification of cell area and length, bright-field images were taken using a Cytation 5 automated cell imaging multimode reader (BioTek Instruments, Winooski, VT, USA) using the 20×, 0.45 NA Plan Fluorite objective. To measure the size of the cells, image analysis was performed in the open-source software CellProfiler (version 4.2.1.). Briefly, non-cellular features in the images were suppressed, and we applied Canny edge detection to enhance cell boundaries. Global thresholding using the minimum cross-entropy was then applied to identify the cells. Overlapping and poorly segmented objects were filtered out, and the area and length of the remaining objects were recorded. The image analysis pipeline that was used to quantify the area and length of the *P. tricornutum* cells using bright-field images is available at Zenodo (DOI: 10.5281/zenodo.5933221): TricornutumSegmentation.cppipe. Examples of the image segmentation used to identify the cells are available in **Supplementary Fig. S6**.

### LL to ML shift time-series experiments

LL-acclimated (0 h) WT and *cpftsy* KO lines were exposed to ML for 0.5, 6, 24 and 168 h. The material was harvested from each line and time point for the pigment analysis, cell count and measurement of photosynthetic parameters as described in the following sections.

### Pigment analyses

Pigment analyses were performed by HPLC according to Rodriguez et al. (2006) using a Hewlett-Packard HPLC 1100 Series system as described previously (Sharma et al. 2021). Pigment values from the HPLC analyses were calculated as femtomol (fmol) pigment per cell. Glutaraldehyde [2% (v/v) final solution] was used for the fixation of cells before determining the cell concentration by flow cytometry as described previously. Cell concentrations were between 0.8 and 2.0 × 10^6^ cells ml^−1^ on the day of harvesting.

### Measurements of photosynthetic parameters

The photosynthetic efficiency (*F*_v_/*F*_m_), the photosynthetic capacity (maximum relative electron transport rate, rETR_max_), the maximum light utilization coefficient (alpha) and the light saturation index (*E*_k_ = rETR_max_/alpha) were calculated based on measurements of variables in vivo Chl *a* fluorescence as previously described (Nymark et al. 2019). The measurements were performed using a PhytoPAM system (System I, Walz, Effeltrich, Germany) equipped with a photomultiplier detector (PM-101P, Walz, Germany) and a Peltier cell (US-T/S, Walz) for temperature control (15°C ± 0.2°C). A strong red LED light (4,000 µmol photons m^−2^ s^−1^, 500 ms, Actinic LED-Array-Cone PHYTO-AL, Walz) was used to provide the saturating pulse. Rapid light curves (from which the rETR_max_, alpha and *E*_k_ were derived) were obtained by exposing the samples to 13 stepwise increasing irradiances (4–1,088 µmol photons m^−2^ s^−1^) at intervals of 30 s each. For the 24- and 168-h samples, an additional step at 1,216 µmol photons m^−2^ s^−1^ was added to ensure that the light saturation plateau was reached. NPQ was induced in LL-acclimated cells by exposure to 470 µmol photons m^−2^ s^−1^ of blue light for 6 min. After 6 min of high-intensity blue-light exposure, NPQ relaxation was measured in very dim light (8 µmol photons m^−2^ s^−1^ of blue light). During the period of NPQ induction and relaxation, *F*_m_ was measured every 30 s using a Multi-Color-PAM (Walz, Germany). All samples were incubated for 3 min in darkness prior to performing measurements.

The pigment band shift (ECS) signal was quantified using a JTS-10 spectrophotometer (BioLogic, Grenoble, France) equipped with interference filters (3- to 8-nm bandwidth) to measure absorption difference signals at selected wavelengths. Actinic light was provided either by an Nd:YAG laser (Minilite II, Continuum, Santa Clara, CA, USA) equipped with a dye cavity to provide saturating red flashes (*l*: 660 nm, intensity: ∼2 mJ, duration: ∼5 ns). Alternatively, continuous light was provided by a red (*l*: 630 nm) LED array. Samples were measured at a concentration of 2 × 10^7^ cells ml^−1^ upon centrifugation of cultures in the exponential growth phase. Measurements of PSII/PSI stoichiometry and electron flow rates were performed as previously described (Bailleul et al. 2010a). The linear component of the ECS (ECS_lin_) (Bailleul et al. 2015) was used to estimate both parameters. This component was deconvoluted from superimposed signals by measuring at three different wavelengths (520, 554 and 563 nm) using the following relationships: Cyt *c* = [554] − 0.4 × [520] − 0.4 × [563], where [554], [520] and [566] were the measured absorption difference signals at the three different wavelengths. ECS_lin_ = [520] − 0.25 × Cyt *c*. PSII contribution was evaluated from the amplitude of the fast ECS_lin_ phase as the decrease in the signal amplitude upon poisoning samples with DCMU (20 µM) and hydroxylamine (HA; 1 mM) to irreversibly block PSII charge separation. PSI was estimated as the fraction of the signal that was insensitive to these inhibitors.

The photosynthetic electron flow (electrons per second) was calculated in steady-state conditions upon exposure to 590 µmol photons m^−2^ s^−1^ of red light. The light was switched off, and the rate of the photosynthetic electron flow was measured as the difference between the slope in the light and the one in the dark as described previously (Nymark et al. 2021). The rate was quantified by dividing the slope difference by the amplitude of the fast ECS_lin_ phase measured after laser exposure in the presence of DCMU and HA, which represents the ECS absorption changes induced by the transfer of a single charge across the membrane (e.g. one electron per photosynthetic chain). Since DCMU and HA block PSII charge separation, electrons are coming from PSI photochemistry.

### HL + LINC exposure experiment

The photosynthetic efficiency (*F*_v_/*F*_m_) was measured in LL-acclimated cells, LL-acclimated cells exposed to HL for 1 h and cells that had recovered from the HL treatment for 30 min. The cells were kept in very dim light during the recovery period. *F*_v_/*F*_m_ was measured using an AquaPen-C (Photon Systems Instruments), and the samples were incubated in darkness for 3 min before measurements. Both the photodamage of PSII and photoprotective mechanism (NPQ) can strongly lower *F*_v_/*F*_m_ during exposure to HL intensities. Additional measurements were therefore performed for cells where LINC (Sigma-Aldrich, St. Louis, MO, USA), an inhibitor of chloroplast protein synthesis (Ridley and Ridley 1979), had been added to the culture 15 min before the start of the HL treatment (final concentration 500 µg ml^−1^). The 15-min incubation period was performed in darkness. LINC blocks PSII repair that depends on the *de novo* synthesis of PSII core components.

### Determining Fe content in *P. tricornutum*

The intracellular Fe content was determined for ML-acclimated *cpftsy.1-25.7, cpftsy.2-4.8* and WT lines (three biological replicates for each cell line). Two different procedures (undiluted and diluted) were employed in order to determine the influence of the excess of Fe present in the culture medium that adsorbed on cell surfaces. For the undiluted procedure, 30-ml cell culture was poured into an acid-washed polycarbonate Nalgene filtration system fitted with a 2-µm (a 47-mm diameter) acid-washed polycarbonate filter (Whatman, Maidstone, United Kingdom). To remove the excess of Fe, 5 ml of oxalate solution was added and incubated for 15 min before filtering at low pressure with a vacuum pump (Tang and Morel 2006, Hassler and Schoemann 2009). The filters were stored frozen until analysis. For the dilution (Dil) procedure, 30 ml of culture was diluted with 60 ml of in-house low-trace-metal artificial seawater and then processed as the undiluted samples. Frozen filters were processed in a high-pressure microwave digestion reactor (UltraCLAVE, Milestone GmbH, Leutkirch, Germany) with a mixture of trace metal grade hydrofluoric and nitric acid (King et al. 2012). After dilution, the samples were analyzed for elemental composition using an 8800 Triple Quadrupole inductive coupled plasma mass spectrometry system (Agilent, Santa Clara, CA, USA) equipped with prepFAST M5 autosampler (ESI, USA) using H_2_ and O_2_ modes.

### Proteomics

Proteomic analyses were performed for *cpftsy.1-25.7, cpftsy.2-4.8* and WT lines that had been acclimated to ML. Five biological replicates were included for each line, and cells were harvested by filtration as described previously (Nymark et al. 2019). Sample preparation and analysis are described in **Supplementary Methods S1**. Briefly, proteins were reduced, alkylated, then cleaned up with hydrophilic interaction liquid chromatography and digested into peptides with trypsin. Peptides were analyzed by LC–MS (C18, 180-min gradient, EASY-nLC 1200, Q Exactive HF). Proteins were identified and quantified by processing MS data using Thermo Scientific™ Proteome Discoverer™ (http://www.thermoscientific.com/content/tfs/en/product/proteome-discoverer-software.html) version 2.5 (PD). Open workflow (Geiszler et al. 2021) provided in FragPipe version 14 from the Nesvizhskii lab (https://fragpipe.nesvilab.org/) was used to inspect the raw files to determine the optimal search criteria. Namely, the following search parameters were used: enzyme specified as trypsin with a maximum of two missed cleavages allowed; acetylation of protein N-terminal including loss of methionine, oxidation of methionine and deamidation of asparagine/glutamine as dynamic post-translational modification, while carbamidomethylation of cysteine as static; and precursor mass-tolerance of 10 PPM, while fragment mass-tolerance of 0.02 Dalton. PD’s node, spectrum files RC, Minora and precursor detector were set up to align/recalibrate, detect features and precursors, respectively. Further internal contaminants database was also queried along with the *P. tricornutum* (strain CCAP 1055/1) translated proteome including isoforms downloaded from PD’s knowledge base (sp_tr_incl_isoforms TaxID = 556484_and_subtaxonomies) in February 2021 (v2021-02-04) using Sequest (Eng et al. 1994) search engines available within the PD ecosystem. For downstream analysis of these peptide-spectra matches (PSMs), both protein and peptide identifications or the PSM FDR was set to 1% as high and 5% as medium confidence; thus, only unique peptides with these confidence thresholds were used for the final protein group identification and to label the level of confidence, respectively. Each protein group abundance was normalized by the total abundance of all identified peptides/PSMs at the FDR mentioned earlier and scaled on all averages with the precursor ion quantifier node of PD. See the Data Availability section for details about deposition of proteomics data to the ProteomeXchange Consortium Perez-Riverol et al. 2019.

### Statistical analysis

To identify significant differences in protein content between *cpftsy* mutants and the WT, the proteomic data were analyzed using the Reproducibility-Optimized Test Statistic package from RStudio (Suomi et al. 2017). Proteins assigned as NA in two or more biological replicates were not included in the analysis. The analysis was run using 1,000 bootstraps, and proteins with *P* < 0.05 and FDR < 0.05 were considered statistically different.

Two-way ANOVA with Dunnett’s multiple comparison tests was carried out using GraphPad Prism software (version 8.4.3) to determine if there were significant differences (*P* < 0.05) between the pigment levels and photosynthetic parameters in *cpftsy* mutants compared to the WT.

## Supplementary Material

pcad014_Supp

## Data Availability

The *CpFTSY* gene has Draft ID Phatr2_14412 and NCBI accession number XM_002182252. The full-length sequence is included in **Supplementary Fig. S1**. UniProt accession numbers for differentially expressed proteins discussed in the text are included in **Table 2**. UniProt accession numbers are provided for all proteins detected by the proteomics analyses, and the entire proteomics data including raw data (raw), peak-list (mzML), identifications (mzID), workflow (pdAnalysis) and annotated results (xlsx) has been deposited to the ProteomeXchange Consortium via the PRIDE (Perez-Riverol et al. 2019) partner repository with the dataset identifier PXD031340. All other relevant information can be found within the manuscript and its supporting information (Perez-Riverol et al. 2019).
