# Supplementary material for: Loss of CpFTSY Reduces Photosynthetic Performance and Affects Insertion of PsaC of PSI in Diatoms
Source: Plant Cell Physiol. 2023 Feb 22;64(6):583–603. doi: 10.1093/pcp/pcad014 (PMC10269134; doi:10.1093/pcp/pcad014)
Supplement: pcad014_Supp [file pcad014_supp.zip › suppl_data/pcp-2022-e-00240-File008.pdf]

Supplemental Material

Methods S1: Sample preparation and proteomic analyses

To each sample, 100 µl of SDC buffer (1% SDC, 10 mM TCEP, 40 mM 2-Chloroacetamide, 100 mM Tris pH 8.5) was added. After mixing by pipetting, contents were transferred to LoBind tubes (Eppendorf), the tubes were kept at 95 °C for 5 min, before being sonicated at 4 °C in a Bioruptor Pico (Diagenode) for 10 cycles, each with 1 min sonication followed by 30 sec without sonication. Protein concentration was measured in a Direct Detect (Millipore) and a volume containing 30 µg total protein was separated for further sample preparation. The volume was adjusted to 50 µl by addition of water. Next, a KingFisher Flex (Thermo Scientific) magnetic-bead handling robot performed clean-up of the proteins using HILIC magnetic microparticles (ReSyn Bioscience) followed by tryptic digestion into peptides. In more details, deep well plates were prepared and placed into the KingFisher as: 25 µl of HILIC microparticles (20 mg/ml) and 175 µl Equilibration buffer (15% Acetonitrile, 100 mM Ammonium acetate pH 4.5) at position 1, 500 µl Equilibration buffer at position 2, 50 µl sample and 50 µl Bind buffer (0.5% SDS, 30% Acetonitrile, 200 mM Ammonium acetate pH 4.5) at position 3, 200 µl 95% Acetonitrile at positions 4 and 5, 50 µl Trypsin (0.1 µg/µl in 45 mM Acetic Acid) and 150 µl Digestion buffer (20 mM Ammonium formate pH 8.2) at position 6, comb Tip at position 7. Then the robot executed the following protocol: picked up the comb tip, collected HILIC magnetic particles, equilibrated HILIC magnetic particles in Equilibration buffer, bound protein to HILIC magnetic particles, washed in high organic content twice, eluted from HILIC and digested protein with Trypsin at 37° C for 4 h, removed HILIC magnetic particles. BindIt Software 4.0 (Thermo Scientific) was used to prepare the robot protocol and to run it. Unless otherwise-stated, chemicals were from Sigma.

Subsequently the digested peptides were transferred to new tubes, the tubes were spun down (16,000g for 15 min) and 100 µl of supernatants were transferred to new tubes. Supernatant was dried at 45°C with a vacuum concentrator. Samples were reconstituted in 70 µl 0.1% Formic Acid by shaking (1000 rpm) at 4°C for 1 h. Then tubes were spun-down (16,000g for 15 min) and 30 µl of supernatants were transferred to LC-MS vials. Analysis was performed on an EASY-nLC 1200 UHPLC system (Thermo Scientific) interfaced with an Q Exactive HF mass spectrometer (Thermo Scientific) via a Nanospray Flex ion source (Thermo Scientific). Peptides were injected onto an Acclaim PepMap100 C18 trap column (75 µm i.d., 2 cm long, 3 µm, 100 Å, Thermo Scientific) and further separated on an Acclaim PepMap100 C18 analytical column (75 µm i.d., 50 cm long, 2 µm, 100 Å, Thermo Scientific) at 250 nl/min using a 180-min gradient (145 min 5%-35% B, 15 min 35%-100% B, 20 min 100% B; where A is 0.1 % formic acid and B is 0.1 % formic acid, 80% acetonitrile). Unless otherwise-stated, chemicals were from Fisher Scientific.

Peptide ions were analysed in positive ion mode under data dependent acquisition using the following parameters: Electrospray voltage 1.9 kV, HCD fragmentation with normalized collision energy 27. Each MS

scan (200 to 2000 m/z, 3e6 AGC target, profile) was acquired at a resolution of 60,000 FWHM, followed by 15,000 FWHM MS/MS scans (200 to 2000 m/z, 1.2 m/z isolation width, 1e5 AGC target, 48 ms maximum IT, centroid) triggered for the 15 most intense ions, with a 25 s dynamic exclusion. Charge exclusion was set to unassigned, 1 and greater than 5.

>PtCpFTSY   Target site 1

MFVVYIVAFLSTPVPVLAFVPSPYRPRFTH**FRTTAAA**GRTSTTELSMVFDFFKERSKEGL 60
GQLEKLKESASRGELGKGLKEAASYTSQTNQAFATGLAKSRNRLLQNIEGLFTGVSPEQV 120
LDDLQDILLQADLGTKTAEDIVAEVKSLREDSTKMLSKDDLKSIMRGKLIEALNTELSGA 180

Target site 2
I**QFSPLVD**KTPTVLFVMGANGMGKTTTIGKLAHRLRNEGNQTVLLAACDTFRAGAVEQLQ 240
QWAERAQVDMVGPSTKVTTPSAVLYAALDKGIAEKYDTILVDTSGRLSNNDQLTAELKKM 300
KKVIQKRLSRENDEEGKPLPNLQVPHETLLVLDAAQGRMALDSAKVWNEEIGLSGLILTK 360
LDGSARGGSVVAISRDIQLPVKLIGVGEGIEDLRDFESERFVDGLLGIGAAGGGSSTSEG 420
AKLAARLKQMRKERDARAKLKKEVSPIGSATSSASVPSNPNQYQAQKPNRPKNKKKKGKK 480

>PtCpFTSY ORF

ATGTTCGTCGTTTACATCGTAGCTTTTCTATCCACACCCGTGCCGGTCCTTGCGTTTGTC   60
CCCTCCCCGTATCGACCAAGGTTCACGCAT**TTCCGTACCACGGCAGCAGCAGG**AAGAACA 120
TCGACTACGGAGTTATCCATGGTCTTTGATTTTTTCAAAGAGCGTTCTAAAGAAGGGTTG 180
GGCCAGCTGGAAAAGTTGAAGGAATCGGCCTCTCGTGGAGAGCTCGGCAAAGGATTGAAA 240
GAGGCGGCGTCCTACACGAGCCAAACCAACCAAGCCTTTGCCACCGGTCTCGCCAAGAGT 300
CGCAATCGCCTACTCCAAAACATTGAAGGACTCTTTACGGGGGTTTCGCCCGAACAAGTC 360
CTGGATGATTTGCAAGATATTCTCTTACAAGCAGATCTCGGAACAAAAACTGCCGAAGAC 420
ATTGTTGCCGAAGTCAAGAGTTTACGAGAGGATTCCACCAAAATGCTTTCCAAGGATGAC 480
TTGAAGAGTATTATGAGGGGTAAACTCATTGAAGCCCTCAATACGGAACTTTCCGGAGCC 540
AT**CCAATTCAGTCCTCTGGTTGACA**AAACACCAACAGTTTTATTTGTCATGGGGGCCAAT 600
GGAATGGGTAAAACTACAACAATCGGCAAACTCGCACATCGTCTCCGCAACGAAGGCAAT 660
CAGACCGTCCTTTTGGCTGCTTGTGATACCTTCCGGGCTGGTGCCGTCGAACAACTCCAA 720
CAATGGGCCGAACGGGCCCAGGTAGATATGGTTGGACCATCCACAAAAGTCACGACACCG 780
AGTGCCGTCTTGTACGCAGCTCTCGACAAGGGTATTGCGGAAAAATACGACACAATTCTG 840
GTCGATACGTCCGGAAGATTGAGCAATAACGATCAACTGACGGCCGAACTAAAGAAAATG 900
AAAAAGGTAATCCAAAAACGTCTTTCACGAGAAAACGATGAGGAAGGTAAACCCTTACCC 960
AACCTTCAAGTACCACACGAAACATTACTCGTTCTAGATGCAGCGCAAGGCCGTATGGCA 1020
CTAGACTCGGCTAAAGTGTGGAACGAAGAAATTGGATTGTCGGGATTAATTCTGACAAAA 1080
CTGGATGGCAGTGCACGGGGAGGCAGCGTGGTGGCAATTAGTCGCGATATACAACTTCCA 1140
GTGAAGCTGATTGGAGTTGGGGAAGGGATTGAAGATTTGCGAGATTTTGAATCAGAACGC 1200
TTCGTTGACGGTCTCCTGGGTATCGGTGCGGCTGGTGGTGGATCGTCGACGAGCGAAGGA 1260
GCCAAGCTAGCGGCGCGGCTGAAGCAAATGCGTAAGGAACGGGACGCTCGCGCTAAACTC 1320
AAAAAGGAAGTCTCGCCGATAGGAAGCGCCACATCATCTGCGTCCGTGCCCAGCAATCCT 1380
AACCAATATCAAGCCCAGAAACCAAATCGGCCGAAAAATAAAAAGAAAAAAGGAAAAAAG 1440
TAA

Supplementary Figure S1. CpFTSY protein and DNA sequences. Target sites are indicated with blue characters and marked with Target site 1 and 2 in the protein sequence.

```
                                                      Amphipathic helix 1              Amphipathic helix 2
                                                          -----------                      -----------
Phaeodactylum tricornutum CpFTSY  : TSTTELSMVFDFFKERSKEGLGQLEKLKESASRGELGKGLKEAASYTSQTN :  90
Amphora coffeaeformis             : TTTTELSMVFDFFRERSQEGVDQLNKLAKAASQGELGKGLSDVAAYTATTN :  98
Asterionellopsis glacialis        : ARNTELNMVFDFFKQRTEEGIEQLSKLSDAASKGNLGQGLAEAAAYTAESN : 107
Amphiprora paludosa               : TGSTKLNMVFDFVRERSKEGLDQLSKLGDAAKVGQLGKGLSEAAAYTSQTN : 130
Fistulifera solaris               : ASKTQLNMVFDFFKDRTKEGIDQLNNLAKSASQGQLGKGLADVASYTNERN :  81
Seminavis robusta                 : QSKTQLFMVFDFFKEKTSEGFDQLNRLADASYKGELGKGISDVAAYTTASN :  90
Cylindrotheca closterium          : SSSTKLPMVFDFFKKRSEEGLDQLSKFSDAASKGDLGKGLADLASYTRETN :  79
Odontella aurita                  : TSTS-LPMVFDFFRKRSSEGLEQLSNLADAASKGKLGEGLVDAASYTQMTN : 121
Grammatophora oceanica            : SSSTQLHMVFDFFKERSKEGVEQLSNLADAAKRGKLGKGLSDAAEYTSETN : 100
Thalassionema frauenfeldii        : KNTQELNMVFDFFKKRSEEGFDQLNNLADAVYKGNIGEGLKDLADYTATTN :  87
Striatella unipunctata            : TSTTQLGMVFDFFKSRAQEGIDQLSNIAEKASQGRLGEALQDSADYTKRTN :  96
Chaetoceros debilis               : HVSTQLH-VFDFFKQRASEGVDQLKNLSEKTAQGKLIEGLSDVGKYTQESN :  87
Fucus ceranoides                  : SGRLGASMLLDFIKKRAQEGVEQTQNLVSAAQQGRLDEALKETSEYVKDRN : 130
Ectocarpus siliculosus            : GSGGGMVMLFDFIKKRAQEGVEQTQNLVTAAQTGRLDEALKETSAYVKDRN : 138
Ochromonas sp. CCMP1899           : TKSSGLYMVFDFLKKRSEEGFAQVSNLAKKTAEGKLGEALGETADYVSSRR :  89
Nannochloropsis gaditana          : RQGESHMMVFDFIRKRAEEGIQQVQNIATKTAEGKFVEALGDTASYVKKRQ : 117
Mallomonas sp.CCMP3275            : -QRTTASMVFDFIKKRSEEGINQFQNIATKSFEGKLGEALSDSALYVRERQ :  83
Epipyxis sp. PR26KG               : -KILKINMVFDFFRQRTQEGIAQVQNIATKTLEGKLEEALRDTSTYIKQRQ :  85
Chattonella subsalsa              : RRNIQVSMVFDFIKKRASEGVSQVSNIAEKAAKGKLGEALSDTGKYIEERQ :  95
Heterosigma akashiwo              : SSRIQTSMVFDFIKKRAQEGLGQASNIAKKTAEGKLGEGLKEAGAYTKET- :  95
Chondrus crispus                  : -RLRTPLAVFDFVRRAGQE---QKKAL-EALREGKGIDYVRDKVKRDIDEV :  86
Pyropia haitanensis               : ------MGVGDFFKRAAKE---RADAW-DALRSGRGLDYAKEKASRDLGTV :  41
Cyanidioschyzon merolae           : GPLGVRAGVFDFLRNFGEQARQEQQALVDAVKQGKFMDYVRDKAVRDAAQV : 142
Cyanidiococcus yangmingshanensis  : GLFALRAGMFDFLRNFGEQARQEQLALLKAMKEGTFMDYVRDKAARDTAQV :  98
```

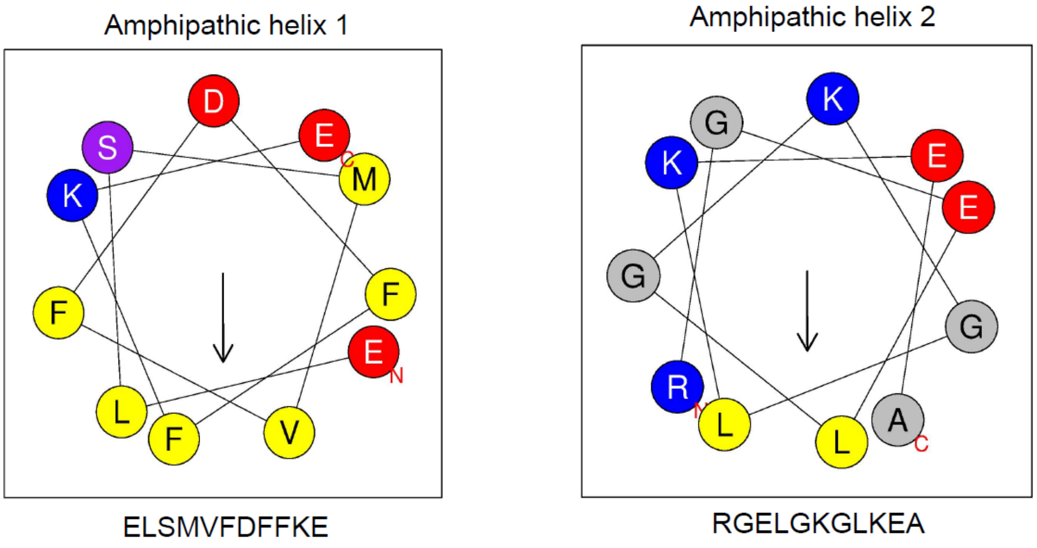


```
                                                                  Polybasic domain
Phaeodactylum tricornutum CpFTSY  : -----------YQAQKPNRPKNKKKK--GKK--  : 480
Asterionellopsis glacialis        : AATASEFEKPSRRRAKPS-NKKKKKKKKKN---  : 513
Amphiprora paludosa               : ---APTFDNDGRPRGNPNRSKKKKKKKKGKN--  : 529
Fistulifera solaris                : -----------NRSPNSRRGTKSKRKKSKK---  : 472
Seminavis robusta                 : ---------TGKSPARRSKPKNKKKKKKGRK--  : 483
Cylindrotheca closterium          : ---AMIETAPANPVSSKNRKKSKKKKNKRKR--  : 476
Odontella aurita                  : ----GGPTGVPRKPKNKNKN-KKRKKRKGKR--  : 546
Grammatophora oceanica            : IITATGTTPSGGRRQGANKSNNKKKKKKKRKRR  : 525
Thalassionema frauenfeldii        : ---------AGRPRMGANSSKKKKKKKRKK---  : 493
Striatella unipunctata            : -------SASPNASNNNKKSKKKKKKKKKIFIY  : 503
Chaetoceros debilis               : -DAITGEDGNPKKTKNKNK-KKKKKKKKGKR--  : 504
Fucus ceranoides                  : ----SYVEPSSKSRKKGRAARGKKSKKKR----  : 533
Ectocarpus siliculosus            : ----------KSRRKTKASKGGKRTKRKKR---  : 522
Ochromonas sp. CCMP1899           : --EVSSLVEGIADNKPKRKKNKPTPKKSKK---  : 484
Nannochloropsis gaditana          : GRGGQGGSSSQRGGGKGGKSGGGKKKRR-----  : 516
Mallomonas sp.CCMP3275            : --------VAKSSRKAVKRVKSKKRQQKSKR--  : 485
Epipyxis sp. PR26KG               : -AFGNDNSPAPNNNMIKKNNRKSKSRRK-----  : 469
Chattonella subsalsa              : --------GPPRGPSGNPRLSRKAKKKRGKR--  : 476
Heterosigma akashiwo              : ------------GGGGGKSSRRKGKKKGGKK--  : 474
```

Supplementary Figure S2. CpFTSY protein alignments including the amphipathic helixes and the polybasic domains.

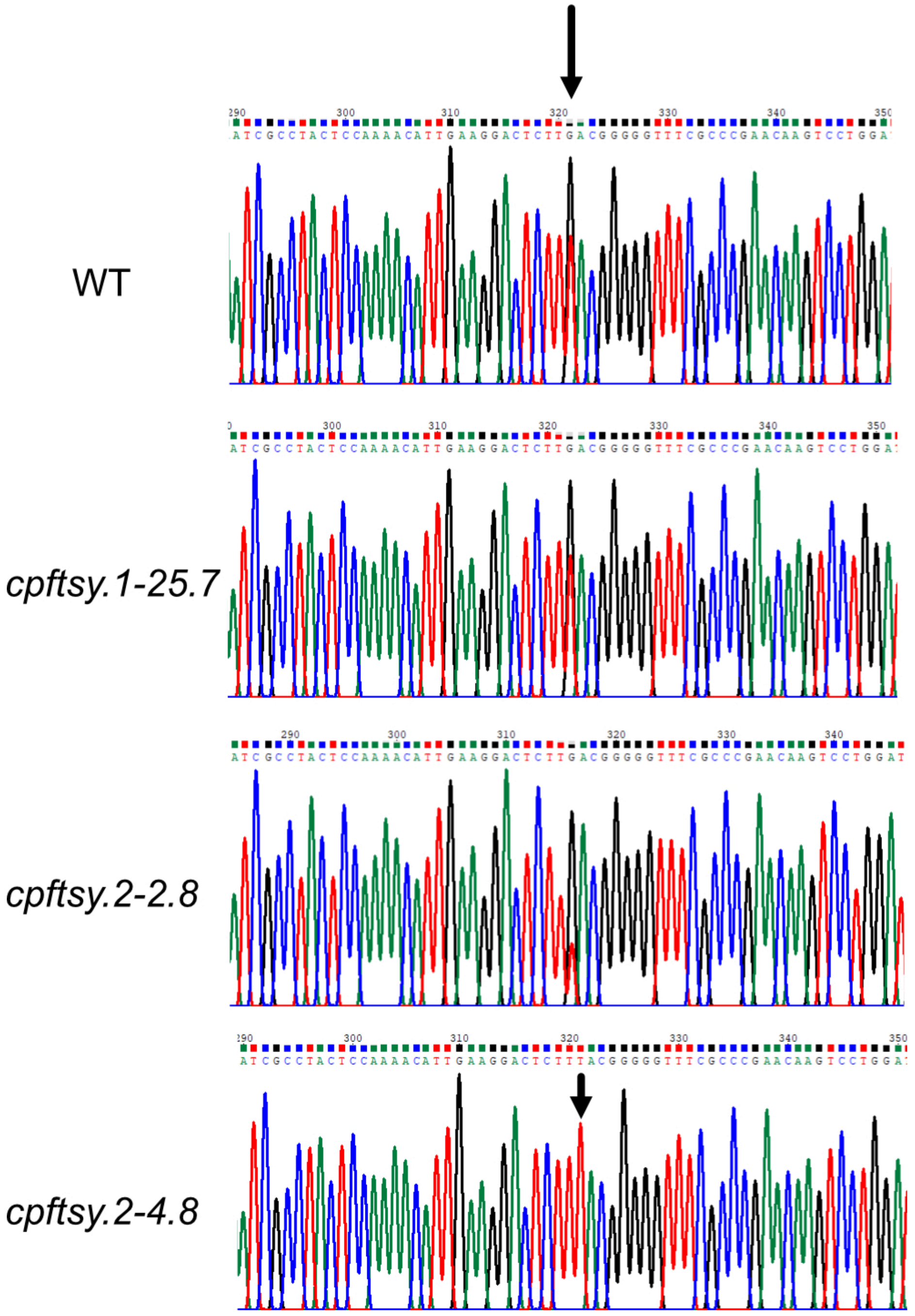

Supplementary Figure S3. Sequence chromatograms showing a polymorphism in the *CpFTSY* gene. Sequences are produced by Sanger sequencing of PCR products from WT and *cpftsy* lines. A G/T polymorphism exists in position 411 (marked by a black arrow) in the *CpFTSY* gene and can be found in WT and two of the three *cpftsy* mutants. The chromatogram resulting from sequencing of *cpftsy.2-4.8* indicates that one allele is lost in this mutant.

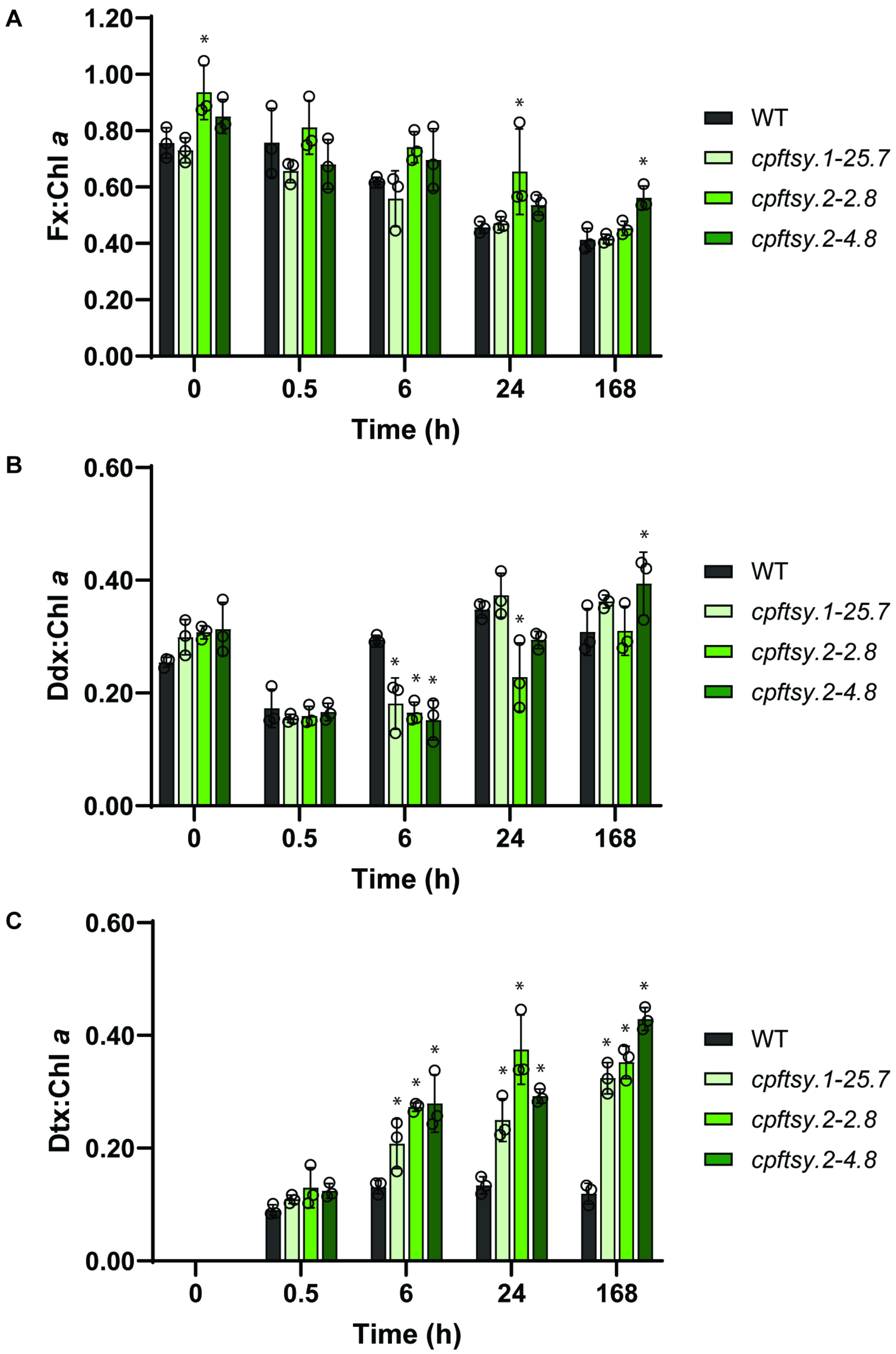

Supplementary Figure S4. Pigments per Chl *a*. A) Fx:Chl *a,* B) Ddx:Chl *a*, C) Dtx:Chl *a* as a function of 0.5, 6, 24 and 168 h of ML exposure time. 0 h time point represents LL acclimated samples. Black circles indicate individual data points for replicates. All results are presented as means of three biological replicates ± SD. Asterisks describe significant differences between c*pftsy* mutants and WT as indicated by two-way ANOVA with Dunnett's multiple comparison test (P < 0.05).

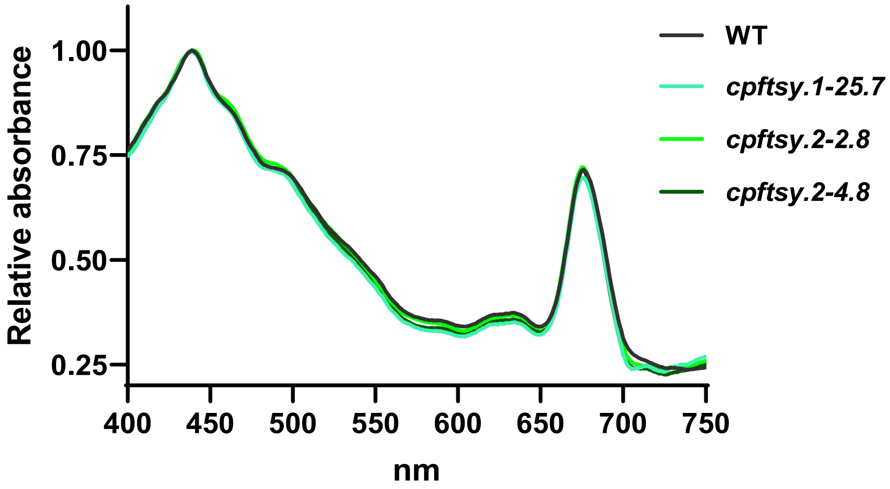

Supplementary Figure S5. Absorbance spectra of WT and *cpftsy* mutant lines. The spectra represent the average of three biological replicates from WT and the three *cpftsy* mutant lines. Cultures were acclimated to LL.

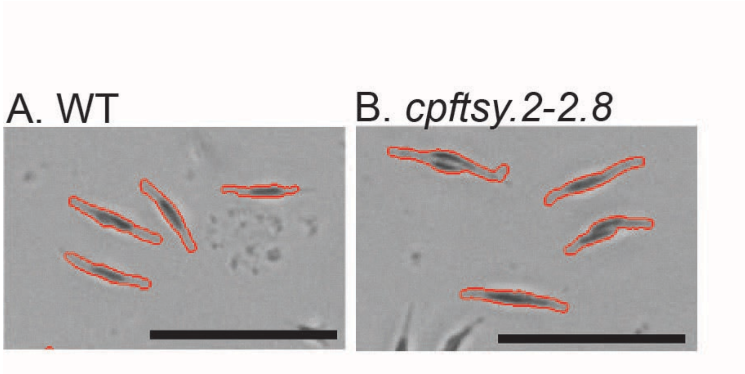

Supplementary Figure S6. Examples of image segmentation, related to Figure 2C. Image segmentation (red) is overlayed onto the brightfield images of for (A) WT and (B) *cpftsy.2-2.8* mutant cells. Scale bar (black) is 50 µm.

Supplemental Table S1. Overview of vector DNA integration sites in the *cpftsy* mutants. Nanopore sequencing of whole genome libraries revealed the vector DNA integrations sites in the *cpftsy* mutant lines. The inserts were only found in one allele, leaving the second allele intact.

	**Integration site 1**	**Integration site 2**	**Integration site 3**
cpftsy.1-25.7	Chr3 (OU594944.1) pos. 1809178, 18S RNA.	Chr8. (OU594949.1) pos. 477403, Phatr3_J19427	Chr8. (OU594949.1) pos. 914719, Phatr3_J40791
cpftsy.2-2.8	Integrated in a highly duplicated genomic region, position is uncertain. Possibly a translocation between Chr21 and another chromosome.<br>Chr21 (OU594962.1) pos. 495411<br>Chr3 (OU594944.1) pos. 1194874<br>Chr23 (OU594964.1) pos. 11480<br>Vector DNA integration in 5' upstream region of Phatr3_J49177 (Chr21)	Integrated in a highly duplicated genomic region, position is uncertain.<br>Chr3. (OU594944.1), pos. 1194874<br>Chr23. (OU594964.1) pos. 532670<br><br>Vector integration in a reverse transcriptase Phatr3_EG00788	
cpftsy.2-4.8	Translocation at PAM target site:<br>Chr14 (OU594955.1) pos. 80803 (FTSY)<br>Chr23 (OU594964.1) pos. 410424.<br>Promoter / 5' UTR of Phatr3_EG00041 (UDP-glucuronate decarboxylase) is fused to FTSY	Chr23 (OU594964.1), pos. 94324, Phatr3_Jdraft420	

Supplemental Table S2. Photosynthetic efficiency (F_v/F_m) during the exponential growth phase of WT and *cpftsy* mutant lines acclimated to LL or ML. The average F_v/F_m during the exponential phase were calculated from three biological replicates of WT and *cpftsy* mutant lines acclimated to LL (35 µmol photons m^{-2} s^{-1}) or ML (200 µmol photons m^{-2} s^{-1}). Values are presented with ±SD.

	35 µmol $s^{-1}m^{-2}$	200 µmol $s^{-1}m^{-2}$
WT	0.72 ± 0.01	0.65 ± 0.01
cpftsy.1-25.7	0.56 ± 0.02	0.53 ± 0.01
cpftsy.2-2.8	0.60 ± 0.02	0.42 ± 0.01
cpftsy.2-4.8	0.58 ± 0.03	0.48 ± 0.02

Supplemental Table S3. Expression data for proteins of the photosynthetic electron transport chain for *cpftsy* lines compared to WT after acclimation to ML (168 h). Ratios were calculated based on results from five biological replicates for each line. Proteins that are significantly regulated in both *cpftsy* mutant lines are marked in bold.

Protein ID	Name	Description	*cpftsy.1-25.7*/WT (log_2 ratio)	FDR	*cpftsy.2-4.8*/WT (log_2 ratio)	FDR	# Unique peptides	# Peptides	Score Sequest HT
A0T0G9	D1 (PsbA)	Photosystem II protein D1	0.90	0.06	0.55	0.27	9	9	256.85
A0T097	D2 (PsbD)	Photosystem II protein D2	0.64	0.22	0.15	0.89	6	6	277.04
A0T0B2	CP47 (PsbB)	Photosystem II CP47 reaction center protein	0.90	0.11	0.43	0.59	20	20	679.31
A0T096	CP43 (PsbC)	Photosystem II CP43 reaction center protein	0.71	0.12	0.15	0.86	16	16	539.35
A0T0A3	PsbE	Cytochrome b559 subunit alpha	0.51	0.18	0.22	0.71	7	7	277.73
A0T0A4	PsbF	Cytochrome b559 subunit beta	**1.08**	**0.02**	-0.02	0.97	1	1	38.11

A0T0A9	PsbH	Photosystem II reaction center protein H	-0.02	0.96	-0.27	0.32	2	2	202.12
A0T0G0	PsbY	Photosystem II protein Y	-0.23	0.52	**-1.40**	**0.01**	1	1	26.76
A0T0H5	Psb28	Photosystem II reaction center psb28 protein	-0.65	0.20	-0.31	0.63	13	13	496.51
B7FZ96	PsbO	Oxygen-evolving enhancer protein 1	-0.41	0.07	**-0.56**	**0.03**	16	16	1041.45
B7G6V4	PsbP	Photosystem II oxygen evolving complex protein PsbP	-0.09	0.90	0.50	0.17	5	5	106.02
B7FZ94	OEE3	Oxygen-evolving enhancer protein 3	-0.49	0.11	**-0.44**	**0.04**	23	23	859.17
A0T0C6	PsbV	Cytochrome c-550	-0.25	0.41	-0.16	0.63	13	13	1302.38
A0T0C9	PetA	Cytochrome f (Cytb$_6$f)	0.30	0.69	0.02	0.98	17	17	412.04
A0T0B8	PetB	Cytochrome b6 (Cytb$_6$f)	0.26	0.74	-0.03	0.98	5	5	112.36
B5Y3C9	PetC2	Cytochrome b$_6$f complex iron-sulfur subunit	-0.08	0.94	-0.17	0.88	7	7	211.34
**A0T0B7**	**PetD**	**Cytochrome b$_6$f complex subunit 4**	**-0.39**	**0.04**	**-0.53**	**0.02**	**3**	**3**	**33.1**
B5Y578	PetJ	Cytochrome c6, cytochrome c553	-0.05	0.92	0.90	0.08	8	8	563
A0T0L9	PsaA	Photosystem I P700 chlorophyll a apoprotein A1	0.30	0.69	0.02	0.98	14	14	531.61
A0T0M7	PsaB	Photosystem I P700 chlorophyll a apoprotein A2	0.38	0.37	0.20	0.76	10	10	208.24
**A0T0L2**	**PsaC**	**Photosystem I iron-sulfur center**	**-1.35**	**0.03**	**-1.96**	**0.01**	**4**	**4**	**176.05**
A0T0B9	PsaD	Photosystem I ferredoxin-binding protein	-0.52	0.13	-0.60	0.10	13	13	493.43
A0T0F3	PsaE	Photosystem I reaction center subunit IV	0.60	0.53	-1.16	0.25	3	3	85.15
A0T0M1	PsaF	Photosystem I protein F	-0.03	0.98	-0.21	0.82	8	8	263.25
A0T0M6	PsaL	Photosystem I reaction center subunit XI	0.50	0.42	0.27	0.78	4	4	179.73
A0T0C8	PetF	Ferredoxin	-1.06	0.22	0.19	0.93	1	1	2.34
A0T0F1	AtpA	ATP synthase subunit alpha	-0.21	0.79	-0.22	0.81	27	24	1382.1
A0T0D2	AtpB	ATP synthase subunit beta	0.10	0.80	0.07	0.90	24	22	1828.61
A0T0F0	AtpD	ATP synthase subunit delta	0.26	0.70	0.20	0.80	7	7	92.87
A0T0D1	AtpE	ATP synthase epsilon chain	-0.45	0.08	-0.32	0.21	8	8	218.07
A0T0E9	AtpF	ATP synthase subunit b	0.44	0.54	0.45	0.57	12	12	168.55
A0T0E8	AtpG	ATP synthase ATP synthase subunit b'	0.26	0.44	0.15	0.69	9	9	186.64

Supplemental Table S4. Cell density. content of phosphorus and iron. and the iron (Fe) to phosphorus (P) ratio of WT and two *cpftsy* mutant lines. The values represent the average of three biological replicates for each cell line. Cultures were acclimated to ML. Stdev: standard deviation. RSD%: relative standard deviation %. Accuracy for the P and Fe content was determined with the Certified reference material form Institute of Nuclear Chemistry and Technology Warzawa - Poland. Oriental Basma Tobacco Leaves (INCT-OBTL-5). Recovery (%) was determined as 87% and 93% for P and Fe respectively.

			Phosphorus (P)			Iron (Fe)					
Treatment	Cell number [cells/ml]	Stdev	Conc. [ug/ml]	Stdev	RSD%	Conc. [ug/ml]	Stdev	RSD%	Fe:P [mmol:mol]	Stdev	RSD%
WT	904930	53833	0.23	0.04	18.6	0.029	0.005	18.3	77.1	1.7	2.2
cpftsy.1-25.7	780135	18165	0.23	0.06	27.0	0.025	0.009	35.2	65.2	5.2	7.9
cpftsy.2-4.8	405001	25428	0.13	0.02	17.0	0.015	0.005	36.1	72.9	15.4	21.1
WT (diluted)	416955	60418	0.05	0.01	12.6	0.005	0.000	5.4	64.1	7.3	11.4
cpftsy.1-25.7 (diluted)	284343	8148	0.04	0.00	6.9	0.001	0.000	5.7	28.0	2.9	10.4
cpftsy.2-4.8 (diluted)	148757	4403	0.03	0.00	12.1	0.002	0.000	17.8	55.2	13.2	23.9

Supplemental Table S5. Oligo and primer sequences. Oligos used to make the adapter inserted into the pKS diaCas9-sgRNA plasmid. PCR primers and HRM primers for screening of *cpftsy* mutants.

Oligo name	Orientation	Sequence (5'-3')	Amplicon size (bp)
CpFTSY_PAM1F	Forward	TCGATTCCGTACCACGGCAGCAGC	-
CpFTSY_PAM1R	Reverse	AAACGCTGCTGCCGTGGTACGGAA	-
CpFTSY_PAM2F	Forward	TCGATGTCAACCAGAGGACTGAAT	-
CpFTSY_PAM2R	Reverse	AAACATTCAGTCCTCTGGTTGACA	-
Pt14412_G1F	Forward	GGCCAGTCTGTAACATGTTCGT	1105
qFTSY_R	Reverse	CTGCCATCCAGTTTTGTCAGAA	
HRM14412.1_F	Forward	CCGTATCGACCAAGGTTCACG	115
HRM14412.1_R	Reverse	CCAACCCTTCTTTAGAACGCTCT	
HRM14412.2_F	Forward	CTCATTGAAGCCCTCAATACGG	131
HRM14412.2_R	Reverse	GCGAGTTTGCCGATTGTTGTAG	
